# Evaluating the Microheterogeneous Distribution of Photochemically Generated Singlet Oxygen Using Furfuryl Amine

**DOI:** 10.1021/acs.est.3c01726

**Published:** 2023-05-02

**Authors:** Kai Cheng, Lizhong Zhang, Garrett McKay

**Affiliations:** † Zachry Department of Civil & Environmental Engineering, Texas A&M University, 3131 TAMU, College Station, Texas 77845, United States; ‡ Department of Physics, University of California, Santa Barbara, Santa Barbara, California 93106, United States

**Keywords:** photochemistry, furfuryl amine, singlet oxygen, dissolved organic matter, microreactor

## Abstract

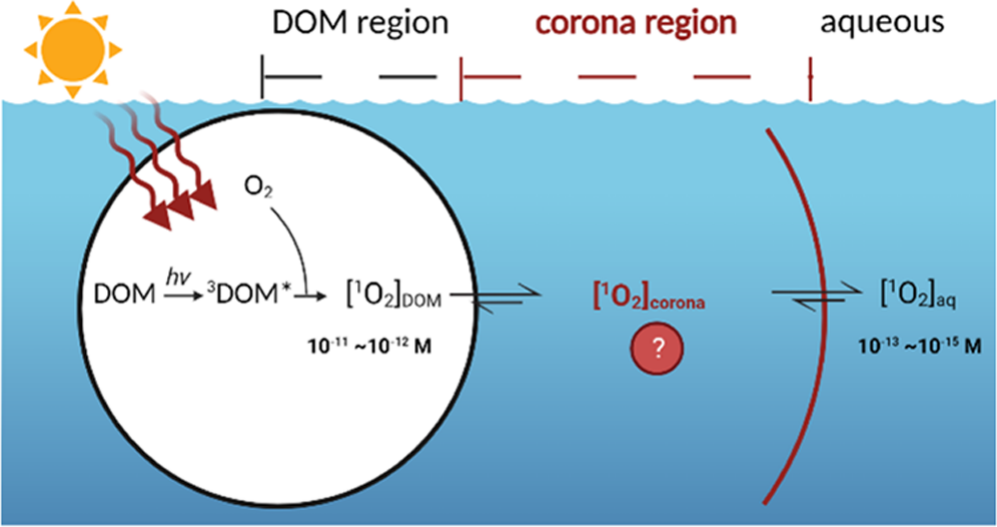

Singlet oxygen (^1^O_2_) is an important reactive species in natural waters produced during photolysis of dissolved organic matter (DOM). Prior studies have demonstrated that ^1^O_2_ exhibits a microheterogeneous distribution, with [^1^O_2_] in the interior of DOM macromolecules ∼30 to 1000-fold greater than in bulk solution. The [^1^O_2_] profile for DOM-containing solutions has been determined mainly by the use of hydrophobic probes, which are not commercially available. In this study, we employed a dual-probe method combining the widely used hydrophilic ^1^O_2_ probe furfuryl alcohol (FFA) and its structural analogue furfuryl amine (FFAm). FFAm exists mainly as a cation at pH <9 and was therefore hypothesized to have an enhanced local concentration in the near-DOM phase, whereas FFA will be distributed homogeneously. The probe pair was used to quantify apparent [^1^O_2_] in DOM samples from different isolation procedures (humic acid, fulvic acid, reverse osmosis) and diverse origins (aquatic and terrestrial) as a function of pH and ionic strength, and all samples studied exhibited enhanced reactivity of FFAm relative to FFA, especially at pH 7 and 8. To quantify the spatial distribution of [^1^O_2_], we combined electrostatic models with Latch and McNeill’s three-phase distribution model. Modeling results for Suwannee River humic acid (SRHA) yield a surface [^1^O_2_] of ∼60 pM, which is ∼96-fold higher than the aqueous-phase [^1^O_2_] measured with FFA. This value is in agreement with prior reports that determined 1–3 orders of magnitude higher [^1^O_2_] in the DOM phase compared to bulk solution. Overall, this work expands the knowledge base of DOM microheterogeneous photochemistry by showing that diverse DOM isolates exhibit this phenomenon. In addition, the dual-probe approach and electrostatic modeling offer a new way to gain mechanistic insight into the spatial distribution of ^1^O_2_ and potentially other photochemically produced reactive intermediates.

## Abstract 2

Singlet oxygen concentrations in the near-surface region of DOM macromolecules were measured using a dual-probe method combining the widely used furfuryl alcohol and its cationic analogue, furfuryl amine.

## Introduction

Dissolved organic matter (DOM) is an important component of aquatic environments. Absorption of sunlight by DOM initiates direct and indirect photochemical reactions by generating reactive intermediates (RIs).^1,2^ Of these RIs, singlet oxygen (^1^O_2_), which is produced via the energy transfer from triplet states of DOM to molecular oxygen, plays an important role in the oxidation of some organic micropollutants (e.g., phenol, N-heterocycles, conjugated dienes, and sulfides), biomolecules, and exogenous inactivation of microorganisms.^3,4^ Furthermore, a recent study has suggested that ^1^O_2_ is involved in the partial photooxidation of DOM, with carboxylic-rich alicyclic molecules identified as major DOM–^1^O_2_ reaction products.^5^

The production of ^1^O_2_ by DOM in natural waters was reported by Zepp et al.^6^ in 1977 using 2,5-dimethylfuran as a probe molecule. Since this report, most environmental photochemistry studies quantify ^1^O_2_ using furfuryl alcohol (FFA) as a probe compound.^7^ However, several studies have demonstrated that the apparent singlet oxygen concentration ([^1^O_2_]_app_) measured using probes of varying hydrophobicity was several orders of magnitude higher than the [^1^O_2_]_app_ measured using FFA. Latch and McNeill^8^ reported intra-DOM ^1^O_2_ concentrations between 30 and 3000-fold greater than the aqueous phase by using a hydrophobic, trap-and-trigger probe, known as the vinyl ether 2-[1-(3-*tert*-butyldimethylsiloxy)phenyl-1-methoxymethylene]adamantane (TPMA). While their initial report was for Aldrich humic acid, a subsequent study by Grandbois et al.^9^ confirmed this behavior for two additional humic substance isolates (Pony Lake fulvic acid and Suwannee River humic acid) using the same hydrophobic TPMA probe. Kohn et al.^10^ investigated the ^1^O_2_ inactivation of MS2 coliphage sensitized by DOM isolates. MS2 was found to associate with DOM, which led to a more rapid inactivation due to the higher [^1^O_2_] at the DOM surface. Chu et al.^11,12^ saw evidence of enhanced ^1^O_2_-mediated transformation of histidine and histamine, which was hypothesized to bind to anionic DOM when these amino acids existed as cations. Other studies have shown that photochemically produced RIs besides ^1^O_2_ also exhibit microheterogeneous concentration profiles. For example, Burns et al.^13,14^ found that hydrated electron-mediated dehalogenation of mirex occurred only when mirex was bound to DOM. Moreover, using chlorinated paraffin as a probe compound, Yan et al.^15^ demonstrated that the hydroxyl radical (^•^OH) also exhibits microheterogeneous distribution with [^•^OH] in the DOM phase being up to ∼200-fold greater than in the aqueous phase. Collectively, these studies have demonstrated the importance of microheterogeneous [RIs] in DOM photochemistry, which may be important in the photochemical fate of hydrophobic and cationic pollutants.^4,16−18^

Despite the advances made in these prior studies, accurate quantification of [^1^O_2_] in the DOM vicinity and how this [^1^O_2_] depends on DOM chemical characteristics remains elusive. Although the microheterogeneous distribution of ^1^O_2_ was successfully described by the use of a hydrophobic TPMA probe and the three-phase model,^8^ TPMA has not been widely adopted, possibly due to its lack of commercial availability. In addition, Grandbois et al.^9^ observed binding of hydrophobic ^1^O_2_ probes to Pony Lake fulvic acid and Suwannee River humic acid; however, no binding was observed for Suwannee River fulvic acid. This means that for DOM samples that show no binding to TPMA, an alternative approach needs to be developed to evaluate the spatial distribution of [^1^O_2_]. More recent microheterogeneous photochemistry studies for ^1^O_2_ and ^•^OH have largely used a single DOM isolate, Suwannee River natural organic matter.^11,12,15^ Thus, how the spatial distribution of [^1^O_2_] depends on DOM physicochemical properties remains poorly characterized. In addition, there have not been significant attempts to elucidate the spatial distribution of [^1^O_2_] since Latch and McNeill’s original report.^8^ For example, Chu et al.^11^ provided evidence of enhanced phototransformation of cationic amino acids but did not explicitly differentiate [^1^O_2_]_DOM_ from [^1^O_2_]_corona_.^19^ Knowledge of the spatial distribution of [^1^O_2_] in the DOM vicinity may provide valuable insights into the photochemical fate of hydrophobic and cationic organic contaminants that could associate with DOM. This information may also aid in ascertaining the role of ^1^O_2_ in DOM photooxidation.^5^

In this study, we employed a dual-probe method^20^ combining hydrophilic FFA and its cationic analogue furfuryl amine (FFAm) to evaluate the microheterogeneous distribution of ^1^O_2_ generated from DOM photolysis. The sensitized photochemical transformation of FFAm was assessed in comparison with FFA under identical solution and illumination conditions. We hypothesized that cationic furfuryl amine (FFAm^+^) would experience enhanced phototransformation due to its electrostatic interaction with negatively charged DOM. To assess the generality of microheterogeneous ^1^O_2_ distribution, FFA and FFAm were applied to a geographically and chemically diverse set of humic substances and natural organic matter isolates. To enable a quantitative understanding of the near-surface [^1^O_2_], electrostatic modeling (ion-impermeable sphere and Poisson–Boltzmann) was combined with Latch and McNeill’s three-phase model.^8^ Using the spatial distribution of [FFAm^+^] surrounding DOM calculated from these models, we were able to determine the apparent [^1^O_2_]_corona_. We then performed a quenching kinetic analysis to derive the concentration gradient of ^1^O_2_ from the surface of DOM. The combination of probe pair results and incorporation of electrostatics into the three-phase model offer a new approach for characterizing microheterogeneous ^1^O_2_ distribution in future studies.

## Materials and Methods

### Chemicals and Solution Preparation

The following reagents were used: perinaphthenone, furfuryl alcohol and furfuryl amine (freshly distilled prior to use), potassium hydrogen phosphate, potassium dihydrogen phosphate, ammonium acetate, acetonitrile, potassium chloride, hydrochloric acid, and sodium hydroxide. Table S1 provides supplier and purity information for all chemicals. DOM isolates studied as the sensitizer in the microheterogeneous system were obtained from the International Humic Substances Society (IHSS), including Suwannee River humic acid (SRHA, 3S101H), Suwannee River fulvic acid (SRFA, 3S101F), Suwannee River natural organic matter (SRNOM, 2R101N), Upper Mississippi River natural organic matter (MRNOM, 1R110N), Elliott Soil humic acid (ESHA, 5S102H), Elliott Soil fulvic acid (ESFA, 5S102F), Pahokee Peat humic acid (PPHA, 1S103H), and Pahokee Peat fulvic acid (PPFA, 2S103F).

All solutions were made using lab-grade water produced from a Barnstead Nanopure purification system (Thermo Scientific, 18.2 MΩ-cm resistivity). The stock solution of perinaphthenone was prepared in methanol at a concentration of 1.51 mM (ε_365_ = 1.02 × 10^4^ M^–1^ cm^–1^). Distilled FFA and FFAm were dissolved in water to achieve stock solutions of 50 mM. Phosphate buffer stock solutions (100 mM) consisting of potassium hydrogen phosphate and potassium dihydrogen phosphate were titrated to pH 4–9 to maintain the pH stability during photolysis. To prepare DOM solutions, solid isolate was dissolved in water with sodium hydroxide added incrementally until a pH of ∼7 was reached, after which the solution was stirred overnight in the dark and subsequently filtered through a 0.45 μm sterile syringe filter (VWR, polyethersulfone). DOM stock solutions with a concentration of ∼200 mg/L were stored at 4 °C and used over a period of several months.

### ^1^O_2_ Formation in Homogeneous and Microheterogeneous Systems

The sensitized photolyses were carried out in both a homogeneous and microheterogeneous experimental setup between pH 4 and 9. Perinaphthenone was employed as the model sensitizer for the homogeneous system with a concentration of 10 μM, and DOM was used in the microheterogeneous system with a concentration of 20 mg/L. The solution (25 mL) used in photolysis experiments was tested with a concentration of FFA or FFAm at 100 μM. Solution pH was buffered with 10 mM phosphate. Samples were irradiated in uncapped, borosilicate glass tubes with a Rayonett merry-go-round photoreactor equipped with mercury vapor lamps of emission maxima at 365 nm (Figure S1). pH and absorbance were monitored at the beginning and end of each irradiation period. Small sample volumes (250 μL) were withdrawn periodically for analysis. Photochemical experiments for each sample at each pH were performed in triplicate. The concentrations of FFA and FFAm were monitored by HPLC to determine an observed first-order rate constant (*k*_obs_). Additionally, to evaluate the impact of ^•^OH on the transformation of FFA and FFAm, methanol was added as a quencher in SRHA-sensitized solution. Results indicate negligible differences in the phototransformation rates of FFAm and FFA in the presence of 100 mM methanol (Figure S2). Details for the analytical methods are provided in Text S1.

Bimolecular rate constants for the reaction of neutral and cationic FFAm with ^1^O_2_ were measured in the perinaphthenone-sensitized system, with FFA as the reference (Text S2). Apparent bimolecular rate constants were calculated between pH 4 and 10 in one-unit increments. Fitting of apparent rate constants as a function of pH was used to determine bimolecular rate constants for neutral and cationic FFAm.

Photolysis experiments in the absence of a sensitizer revealed no significant direct phototransformation of FFA and FFAm (Table S2). Additionally, control experiments were conducted to assess the participation of triplet-state sensitizer in the phototransformation of FFA and FFAm. Solutions consisting of perinaphthenone (10 μM) or SRHA (20 mg/L) and the probe pair were prepared and transferred to borosilicate glass tubes equipped with gastight caps. Nitrogen was used to purge the solutions for around 3 min before subjecting the tubes to irradiation. Both FFA and FFAm showed only minor decreases in concentration at the first kinetic time point (<10%) and remained unaltered throughout the rest of the experiment (Text S4 and Figure S3). This observation is consistent with some residual dissolved oxygen remaining in the system, as it is well known that N_2_ sparging does not completely remove dissolved oxygen.

### Ion-Impermeable Sphere Model

Several models^21^ have been used to describe the interaction of cations with humic substances. In this work, an ion-impermeable sphere model was selected to enable the Poisson–Boltzmann-based delineation of the spatial dependence of DOM’s electrostatic potential and the resulting accumulation of cations within DOM’s corona region. Ion-impermeable models consider DOM as a monodisperse, impermeable particle in spherical geometry^22^ with the electrical potential evenly distributed on the surface.^23^ The key variable for calculating the Coulombic effect is the electrostatic potential (φ), usually obtained as the solution of the Poisson–Boltzmann equation (eq 1).
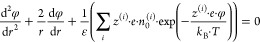
1The equation is composed of two parts: the Poisson equation in spherical geometry with *r* representing the radial distance to the center of a DOM molecule and the Boltzmann distribution of ions responding to the electric field. Here, φ is the electrical potential, ε is the absolute permittivity of the solution, *e* is the elementary charge, *n*_0_^(*i*)^ indicates the concentration of ion *i* in bulk solution with a charge number of *z*^(*i*)^, *T* is the solution temperature, and *k*_B_ is the Boltzmann constant.

To solve the Poisson–Boltzmann equation, it is necessary to know both the concentration of ions in the bulk solution and the electric potential at the surface of the DOM (φ_s_). The latter is calculated from the overall charge density as a function of solution pH. Similar to past studies, the modified Henderson–Hasselbalch equation was used to model DOM charge density by assuming two major classes of proton binding sites (eq 2).^11,24,25^
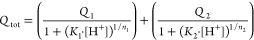
2

where *Q*_tot_ represents the overall negative charge density of DOM, while *Q*_1_ and *Q*_2_ refer to the charge density of specific groups within the DOM, which are often associated with carboxylic acids (*Q*_1_) and phenols (*Q*_2_). Binding constants and their distribution for each group are represented by *K* and *n*, respectively. By assuming that φ is created by the central charged region of DOM molecule, φ_s_ can be determined based on DOM radius, which is the single adjustable parameter that is estimated from molecular size (*M*_n_) determined by size exclusion chromatography measurements (Table S8).^26^ A numerical approach was developed for the nonlinear equation using a successive approximation method.^23,27,28^ The solution to Poisson–Boltzmann equation describes the charge profile of a DOM molecule and the altered ion distribution resulting from the ensuing electrostatic potential. Additional details of the calculation are provided in Text S7.

## Results and Discussion

### Characterization of FFAm as a ^1^O_2_ Probe Compound

**Figure 1:**
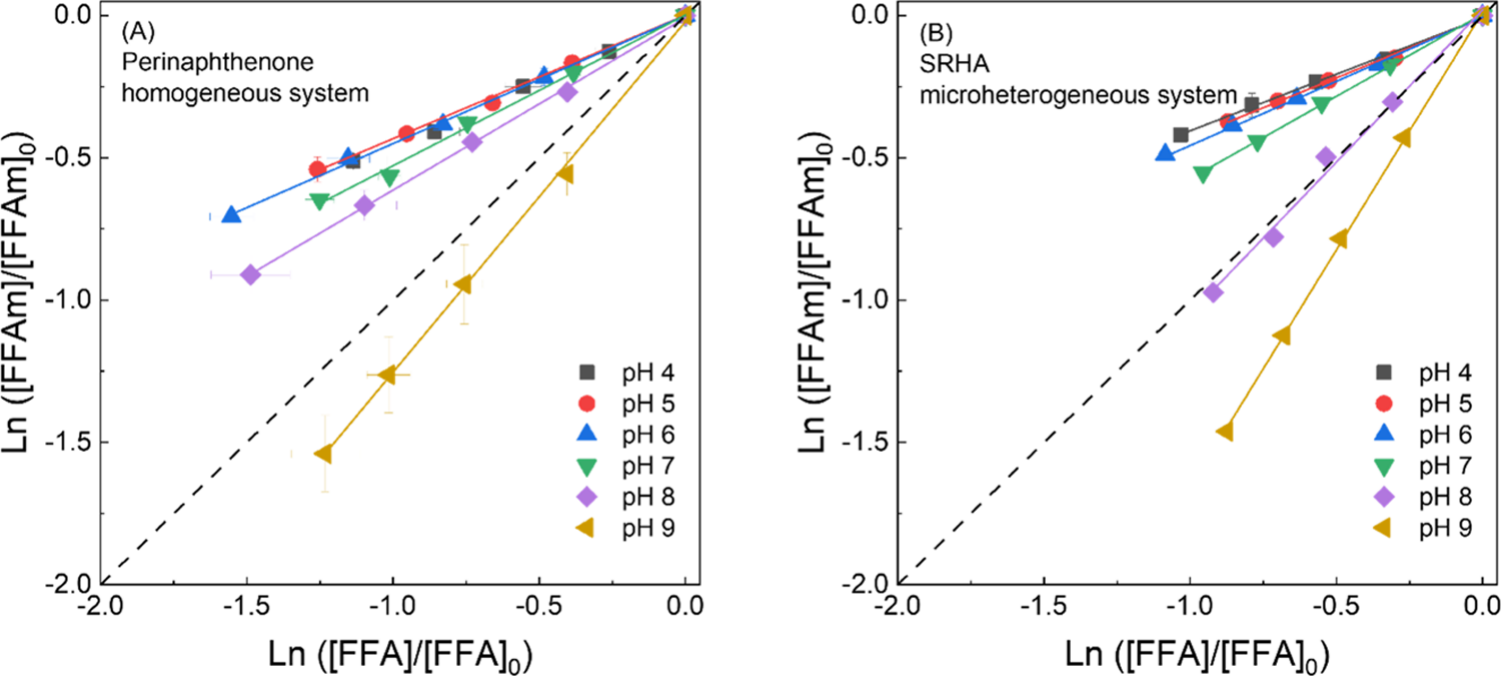
Singlet oxygen-mediated phototransformation of furfuryl alcohol (FFA) and furfuryl amine (FFAm). (A) Perinaphthenone (10 μM) used as a sensitizer with pH ranging from 4 to 9. (B) SRHA (20 mg/L) used as a sensitizer with pH ranging from 4 to 9. All solutions were buffered by 10 mM phosphate. FFA or FFAm was spiked into the solution at the concentration of 100 μM. Irradiation was operated under UV lamp at 365 nm at room temperature (∼21 °C). Solid lines represent the first-order fitting of the experimental data.

Photolysis experiments using FFA and FFAm as ^1^O_2_ probes were conducted between pH 4 and 9 using either perinaphthenone (10 μM) or SRHA (20 mg/L) as ^1^O_2_ sensitizers. When using perinaphthenone as the sensitizer (Figure 1A), transformation kinetics of FFA versus FFAm resulted in linear logarithmic plots, with slopes representing the ratio of first-order rate constants (Figure S5 shows first-order photodegradation rate constants). For the perinaphthenone-sensitized system, the ratio of reaction rate constants between FFAm and FFA (*k*_obs_^FFAm^/*k*_obs_^FFA^) increased from pH 4 to 8, ranging from 0.43 ± 0.04 to 0.60 ± 0.07, which indicates that FFAm has a lower reactivity with ^1^O_2_ compared to FFA. At pH 9, *k*_obs_^FFAm^/*k*_obs_^FFA^ increased to 1.23 ± 0.15. These results are consistent with an increased reactivity between deprotonated FFAm and electrophilic ^1^O_2_ versus protonated FFAm.^4^ FFA is a well-characterized probe compound whose reactivity with ^1^O_2_ shows no pH dependence.^29^ FFAm-^1^O_2_ rate constants were determined by using FFA as a reference compound with a known FFA-^1^O_2_ rate constant (1.0 × 10^8^ M^–1^ s^–1^).^29^ Apparent rate constants of for the reaction of FFAm with ^1^O_2_ as a function of pH can be found in Text S2 and Table S3. Furthermore, the apparent bimolecular rate constants of FFAm as a function of pH were fitted to the ion speciation equation, resulting in a p*K*_a_ of FFAm of 9.01 ± 0.07 (versus 8.89 by Williams et al.^30^). In contrast, when SRHA was used as the sensitizer (Figure 1B), *k*_obs_^FFAm^/*k*_obs_^FFA^ progressively increased from 0.40 ± 0.02 to 1.67 ± 0.02 between pH 4 and 9. The results suggest that ^1^O_2_-mediated transformation of FFAm relative to FFA occurs to a greater extent in DOM than perinaphthenone at neutral to alkaline pH. Control experiments described above showed negligible transformation of FFA or FFAm in N_2_-saturated solution solutions of both SRHA and perinapthenone, suggesting that ^1^O_2_ (and not ^3^DOM*) is the main reactive species responsible for FFAm and FFA degradation.

### Enhancement Factor

To quantitatively evaluate the relative reactivity of FFAm and FFA, the ratio of *k*_obs_^FFAm^/*k*_obs_^FFA^ between the DOM and the perinaphthenone system is defined as the enhancement factor (EF), which represents the apparent [^1^O_2_] experienced by FFAm relative to FFA (eq 3).
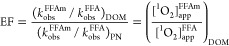
3

In the homogeneous perinaphthenone-sensitized system, FFA and FFAm are exposed to the same [^1^O_2_]. Conversely, in the microheterogeneous DOM system, our results indicated that the [^1^O_2_] was nonuniformly distributed, with higher concentrations near DOM than the bulk solution. Therefore, an EF > 1 implies that FFAm experiences a higher apparent [^1^O_2_] than FFA in DOM-containing solution. If EF = 1, FFAm is exposed to the same concentration of ^1^O_2_ as FFA in the DOM system (Text S3).

An important assumption in eq 3 is that the FFAm-^1^O_2_ bimolecular rate constant in DOM-containing solution is equivalent to that in perinaphthenone solution. For example, if FFAm and a DOM carboxyl group exist as ion pair, the electron density of the furan moiety may be increased, resulting in a faster bimolecular rate constant with ^1^O_2_. To examine the potential impact of ion pairing on FFAm reactivity, we added an increasing concentration of sodium formate from 50 to 1000 μM in the perinaphthenone-sensitized system at pH 8. Table S4 indicates that the reactivity of FFAm was unaffected by the presence of formate up to 1000 μM. This result suggests either that such ion pairs did not form in solution to an extent that would impact FFAm transformation or that, if the ion pair formed, it has the same reactivity as free furfuryl amine.

For SRHA, EF ranged between 0.93 ± 0.05 (at pH 4) and 1.86 ± 0.05 (at pH 8) and decreased to 1.35 ± 0.02 at pH 9. EF > 1 indicates that ^1^O_2_ has a nonuniform geometric distribution around SRHA such that FFAm experienced a higher [^1^O_2_]_app_ than FFA at neutral to alkaline pH. Prior studies have also demonstrated higher [^1^O_2_]_app_ measured by hydrophobic (TPMA) and cationic compounds (histamine and histidine). For example, [^1^O_2_]_app_ for SRHA at an equivalent DOM concentration (∼20 mg/L) quantified by TPMA (∼1.0 pM) was ∼10-fold higher than quantified by FFA (∼0.12 pM after correction for light screening).^9^ Chu et al.^11^ also reported enhancements for SRNOM-sensitized degradation of histidine by ^1^O_2_, with [^1^O_2_]_app_ being 4-fold higher at pH 4, 2-fold higher at pH 5, and equivalent at pH 6. Our study found that the [^1^O_2_]_app_ experienced by FFAm in SRHA-containing solution is 1.86-fold greater at pH 8 than [^1^O_2_]_app_ experienced by FFA. The increased reactivity of FFAm is believed to be caused by the association of FFAm with SRHA, which apparently showed a maximum at pH 8. Before presenting an explanation for this pH dependence (Qualitative Explanation of Enhancement Factor), we first present results from experiments aimed to ascertain the nature of the associations between SRHA and FFAm.

### Kinetic Solvent Isotope Effect

Photolysis experiments were performed in D_2_O to test the hypothesis that the enhanced FFAm phototransformation results from an increased local concentration of FFAm in the DOM vicinity. Experiments were performed using 99.9% D_2_O as the solvent and SRHA (20 mg/L) as the sensitizer with pD equal to 8.0. As shown in Table S5, the observed rate constants (*k*_obs_) for FFA and FFAm photodegradation were both faster in D_2_O due to the increased [^1^O_2_]. For FFA, the relative reactivity in D_2_O versus H_2_O was *k*_obs_^D_2_O^/*k*_obs_^H_2_O^ = 13.5. This is in agreement with the conclusion that [^1^O_2_]_app_ measured by hydrophilic probes in D_2_O should be higher by a factor of 13 than measured in H_2_O.^8^ The enhancement is less than the theoretical 18.6 (based on ^1^O_2_ lifetimes in pure D_2_O versus H_2_O) due to air–D_2_O transfer of H_2_O. By contrast, *k*_obs_^D_2_O^/*k*_obs_^H_2_O^ for FFAm increased by only a factor of 4.6. The lower value of *k*_obs_^D_2_O^/*k*_obs_^H_2_O^ for FFAm suggests that a fraction of FFAm^+^ exists near DOM molecules, reacting close to the source of ^1^O_2_ before significant solvent quenching can occur. Note that control experiments in deaerated solution suggest that FFAm is not appreciably degraded by triplet-state DOM (Figure S3). Kohn et al.^10^ also observed a 5-fold kinetic solvent isotope effect for ^1^O_2_-mediated inactivation of MS2 bacteriophage in SRHA-sensitized solution; FFA’s degradation rate constant was enhanced substantially more in D_2_O compared to MS2′s inactivation rate constant, which was attributed to virus-DOM associations. Conversely, previous studies using hydrophobic probes observed minimal to no change in probe degradation rate constants in D_2_O, with kinetic solvent isotope effects ranging from 0.9 to 1.2.^8,20^ As the isotope effect for FFAm is less than FFA but greater than hydrophobic probes, it is reasonable to suggest that FFAm reactions with ^1^O_2_ occur to a greater extent in the near-DOM vicinity than in bulk solution. These results from D_2_O experiments corroborate the EF approach presented above using perinaphthenone and DOM as ^1^O_2_ sensitizers.

### Comparison of Enhancement Factors between DOM Samples

#### pH Dependence

**Figure 2:**
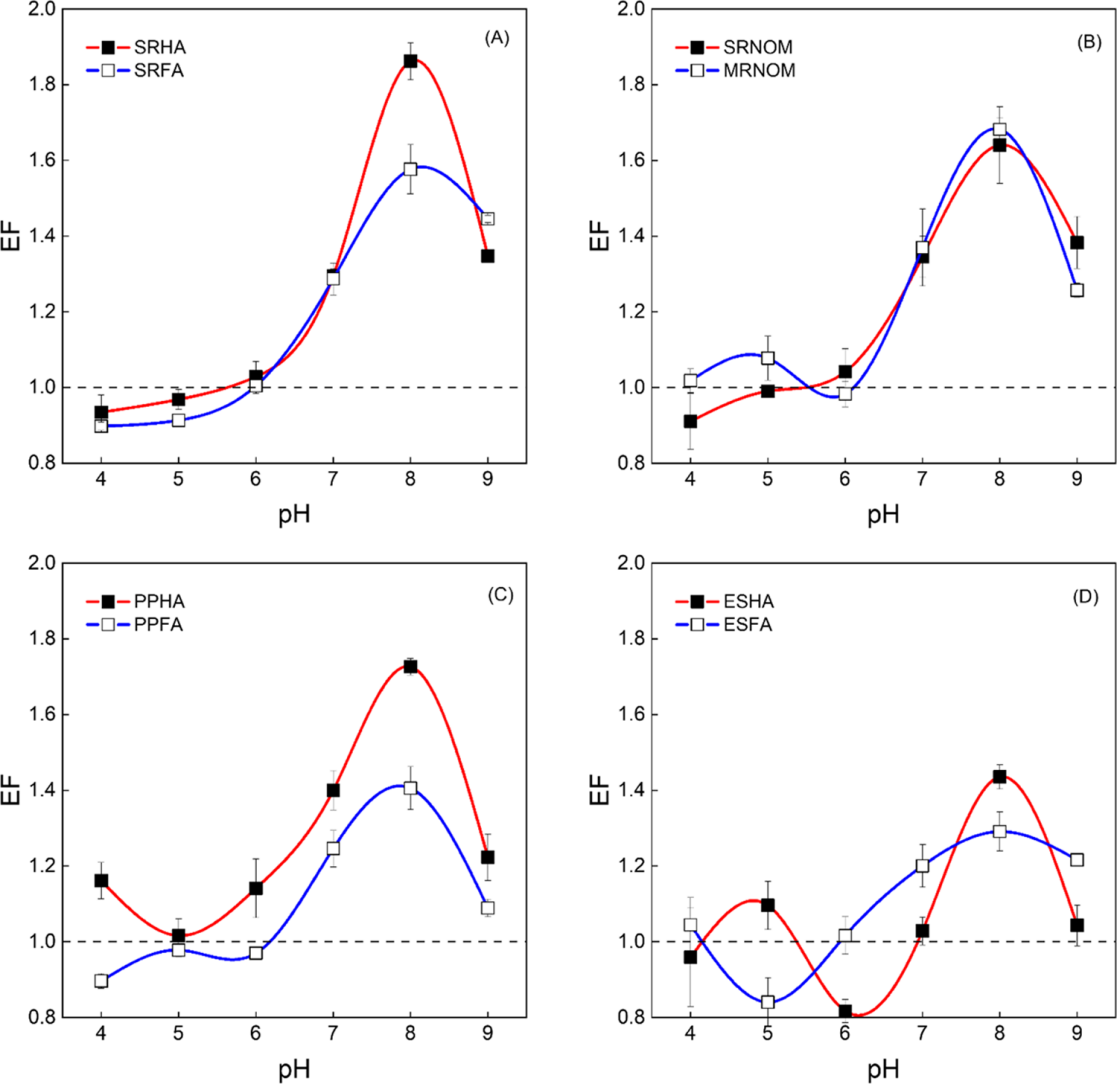
Enhancement factor (EF) plotted as a function of pH for DOM isolates from aquatic and soil sources. Irradiation for all DOM isolates was performed under UV lamp at 365 nm wavelength at room temperature (∼21 °C). (A) SRHA and SRFA isolates. (B) MRNOM and SRNOM isolates. (C) PPHA and PPFA. (D) ESHA and ESFA isolates. All isolates were prepared at a concentration of 20 mg/L and buffered by 10 mM phosphate. EFs in the figure were determined by eq 3 using the slope ratio from DOM system and modeled-corrected slope ratio from the perinaphthenone system (Tables S3 and S6).

To assess the generality of microheterogeneous ^1^O_2_ distribution, the FFAm and FFA probe compounds were applied to a collection of chemically and geographically diverse DOM samples, which all showed enhanced phototransformation of FFAm compared to perinaphthenone between pH 6 to 9 (Figure 2). At pH 8 (the EF maximum), higher EF values were observed for humic acids compared with their fulvic acids counterparts, such as SRHA (1.86 ± 0.05) versus SRFA (1.58 ± 0.06), PPHA (1.73 ± 0.02) versus PPFA (1.41 ± 0.06), and ESHA (1.44 ± 0.03) versus ESFA (1.29 ± 0.02). SRNOM and MRNOM represent ∼90% of the DOM content of their respective source waters compared to the humic substance fraction, which represents ∼50% of the dissolved organic carbon concentration.^31^ SRNOM and MRNOM exhibited similar EF values of 1.64 ± 0.10 and 1.68 ± 0.03 at pH 8, respectively. SRNOM consists of more fulvic acid fraction than humic acid, and the EF behavior displayed more resemblances to SRFA as a result.^32^

Although there are differences in EF values among the isolates, these values alone do not reveal differences in the extent of ^1^O_2_ microheterogeneity because the measured *k*_obs_^FFAm^ values from which EF values are calculated depend on the spatial distribution of [^1^O_2_] and [FFAm^+^]. In terms of [^1^O_2_], DOM size, structure, and abundance of various functional groups^7,33,34^ could all be expected to impact the microheterogeneous distribution of ^1^O_2_. The distribution of [FFAm^+^] can be conceptualized as being due to the electrostatic attraction between FFAm^+^ and negatively charged DOM, whose charge density relies on the chemical composition and molecular conformation. Efforts to determine the spatial dependence of [^1^O_2_] are described in the Qualitative Explanation of Enhancement Factor for [^1^O_2_] Microheterogeneity section.

#### Ionic Strength Dependence

**Figure 3:**
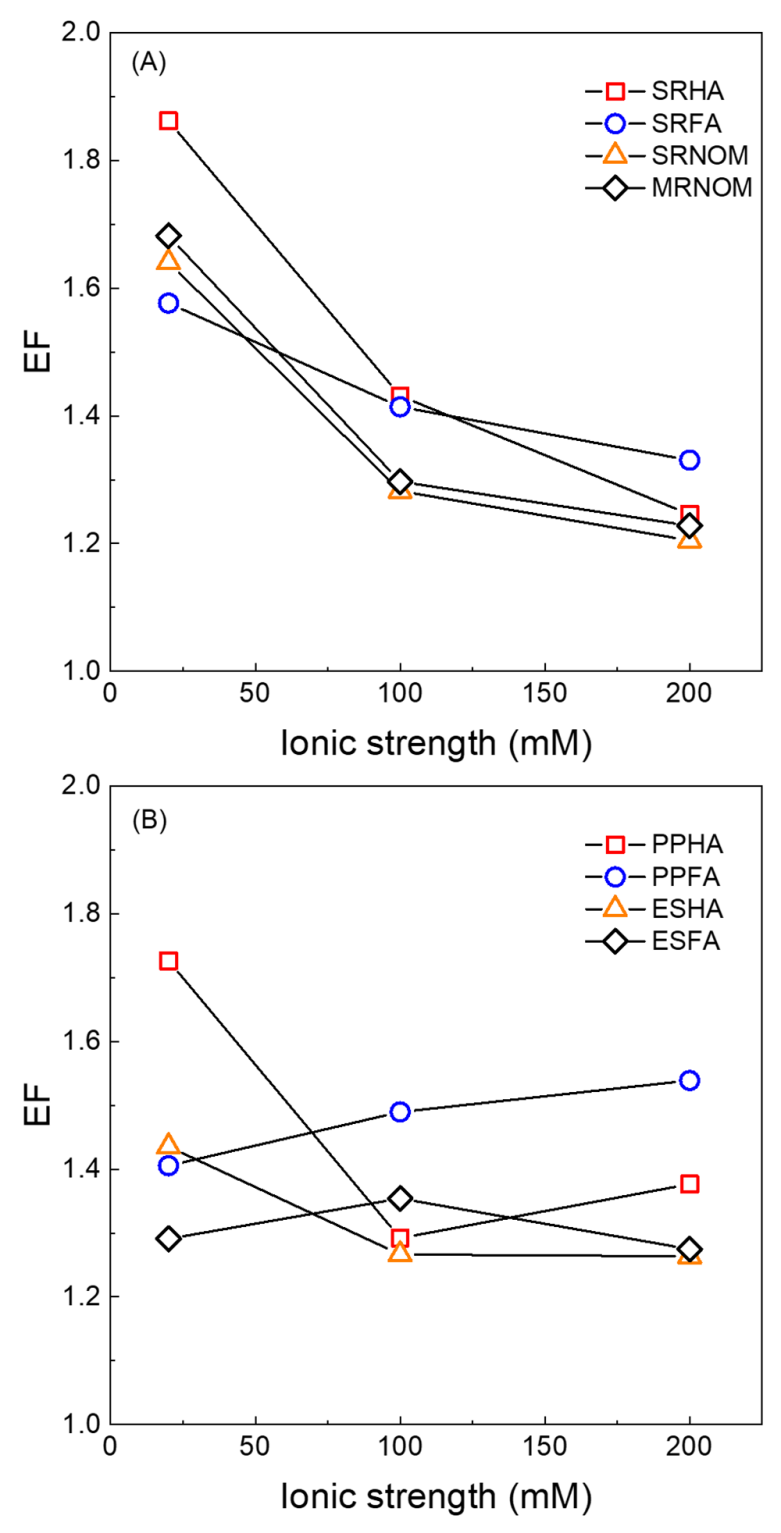
Dependence of enhancement factor (EF) on ionic strength for (A) aquatic and (B) soil isolates at pH 8. Solution pH was buffered by 10 mM phosphate. Ionic strength was controlled by potassium chloride.

Increased ionic strength is expected to reduce the accumulation of FFAm in the DOM vicinity and therefore the measured EF.^35−37^ EF values measured at pH 8 decreased with increasing ionic strength for all aquatic DOM isolates but to different levels (Figure 3). When ionic strength was increased from 20 to 200 mM, the EF of SRHA at pH 8 was reduced from 1.86 to 1.25. For SRHA, *k*_obs_^FFA^ was minimally affected by ionic strength, decreasing from 0.225 to 0.201 h^–1^, whereas *k*_obs_^FFAm^ decreased from 0.242 to 0.145 h^–1^ (see Table S7). Of the aquatic isolates, SRFA exhibited the least change in EF between 20 and 200 mM. The dependence of EF on ionic strength was similar for MRNOM and SRNOM. Overall, the results for aquatic DOM isolates (Figure 3A) support the hypothesis that an increase of ionic strength would exert a shielding effect on ionized molecules and decrease the electrostatic attraction between FFAm^+^ and DOM.

Results for terrestrial isolates (PPHA, PPFA, ESHA, and ESFA) are more varied (Figure 3B). EF values for PPHA and ESHA decreased with increasing ionic strength, but values for the corresponding fulvic acid isolates remained the same or increased slightly. *k*_obs_^FFA^ increased with increasing ionic strength for PPHA but decreased for PPFA, ESHA, and ESFA. *k*_obs_^FFAm^ increased with increasing ionic strength for PPHA but again decreased for PPFA, ESHA, and ESFA. Possible explanations for these results include a greater impact of ionic strength on the three-dimensional conformation of soil humic substance isolates, a difference in interaction mechanism between FFAm and soil isolates (e.g., inner-sphere complexes) compared to aquatic isolates, or some combination of these factors. Future research is required to more fully comprehend the difference in the behavior of EF with varying ionic strength for soil isolates.

#### Qualitative Explanation of Enhancement Factor

According to the Boltzmann distribution equation (eq 1), the enhanced local concentration of FFAm^+^ in the DOM vicinity is determined in part by DOM’s surface potential. As pH increased from 4 to 8, the charge density increased by nearly 2-fold for all isolates (see Table S9), while the fraction of FFAm^+^ was relatively unchanged (α_0_ > 0.9). For example, using the *M*_n_ values in Table S8 to calculate the average number of charges per DOM “molecule,” the charge density of SRHA increased from 5.44 to 11.70 equiv/mol from pH 4 to 8. Therefore, the increase in EF from pH 4 to 8 results from the increase in DOM’s charge density due to the increasing fraction of deprotonated carboxylic acids and phenols (Figure S7). The higher surface potential that results from increased deprotonation of these groups is expected to attract an increasing concentration of FFAm^+^ near the DOM vicinity compared to its analogous neutral compound FFA. The enhanced [FFAm^+^] in the DOM corona region increases the [^1^O_2_]_app_ sampled by FFAm^+^. The decrease in EF between pH 8 and 9 corresponds to the increasing fraction of neutral FFAm in solution (measured p*K*_a_ of 9.0).

This qualitative explanation is in agreement with previous studies of organic cation or zwitterion interactions with DOM.^24,38,39^ For example, histidine and histamine exhibited enhanced reactivity in SRNOM solution at pH < 6 (where histidine and histamine existed as a cationic species) but little to no enhanced reactivity for histidine at pH > 6 (where the molecule had an overall neutral charge) and minor enhancement for histamine (net positive charge decreased from two to one at pH > 6).^11^ One key difference between our study and that of Chu et al.^11^ is that no ionic strength effect was observed for histamine and histidine, suggesting that the enhanced ^1^O_2_-mediated photodegradation observed for these amino acids was due to inner-sphere interactions. In contrast, the EF variation with ionic strength observed for FFAm suggests that accumulation near DOM is driven by nonspecific electrostatic attractions.

### Quantitative Models for [^1^O_2_] Microheterogeneity

#### Multiphase Model to Quantify FFAm–DOM Interaction

The above section presented a qualitative assessment of the factors governing FFAm transformation. To provide quantitative information, it is necessary to apply mathematical models that make some simplifying assumptions, most importantly that DOM is composed of monodisperse spherical particles.

We first employed the three-phase model developed by Latch and McNeill.^8^ Treating DOM in spherical geometry leads to three regions of [^1^O_2_]: the interior hydrophobic DOM phase with the highest [^1^O_2_], an aqueous ^1^O_2_-containing corona region surrounding the DOM phase, and the bulk water region. To adapt this model to our system, we assume, like others, that quenching of ^1^O_2_ by DOM is much slower than diffusive loss^9^ and that FFAm can exist only in the aqueous phase (DOM corona region and bulk solution) but not in the DOM phase (the latter is a consequence of applying the ion-impermeable model). Under these assumptions, the three-phase model can be simplified to the corona and aqueous phases, yielding the observed first-order phototransformation rate constant of FFAm (*k*_obs_^FFAm^) as eq 4
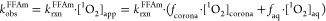
4where *k*_rxn_^FFAm^ is the bimolecular rate constant of FFAm with ^1^O_2_ and *f* is the fraction of FFAm in either the bulk aqueous phase or corona. To calculate [^1^O_2_]_corona_, it is necessary to have *k*_obs_^FFAm^, [^1^O_2_]_aq_, *f*_corona_, and *f*_aq_. *k*_obs_^FFAm^ was determined from photochemical experiments. A reasonable upper bound estimate for the [^1^O_2_]_aq_ experienced by FFAm is determined by the [^1^O_2_]_ss_ quantified using FFA. The actual bulk aqueous-phase [^1^O_2_] in FFAm-containing solution may be lower, as the elevated concentration of FFAm^+^ in the DOM corona phase leads to increased quenching and thereby reduces the escape of ^1^O_2_ to the bulk phase. The remaining parameters (*f*_corona_ and *f*_aq_) are calculated through electrostatic modeling.

#### Electrostatic Modeling to Calculate [FFAm^+^]

We used the Poisson–Boltzmann model for calculating the geometric distribution of FFAm^+^ from DOM as a function of distance, thereby permitting calculation of *f*_corona_ (see Text S8). This approach assumes that the partitioning of FFAm^+^ between the corona and bulk phase is dominated by electrostatic effects, that φ is generated from a spherical point charge, and that φ_s_ (surface potential) can be determined based on DOM radius. As molecular weight is proportional to *R*^3^, the radius can be obtained from DOM’s density and molecular weight. While these calculations can, in principle, be performed for all DOM samples, the input parameters (e.g., molecular weight) are not well constrained. Therefore, we focus on SRHA in the below calculations as a radius of 1 nm (see Text S5), which has been used for this isolate in several previous studies.^9,15,40−42^

**Figure 4:**
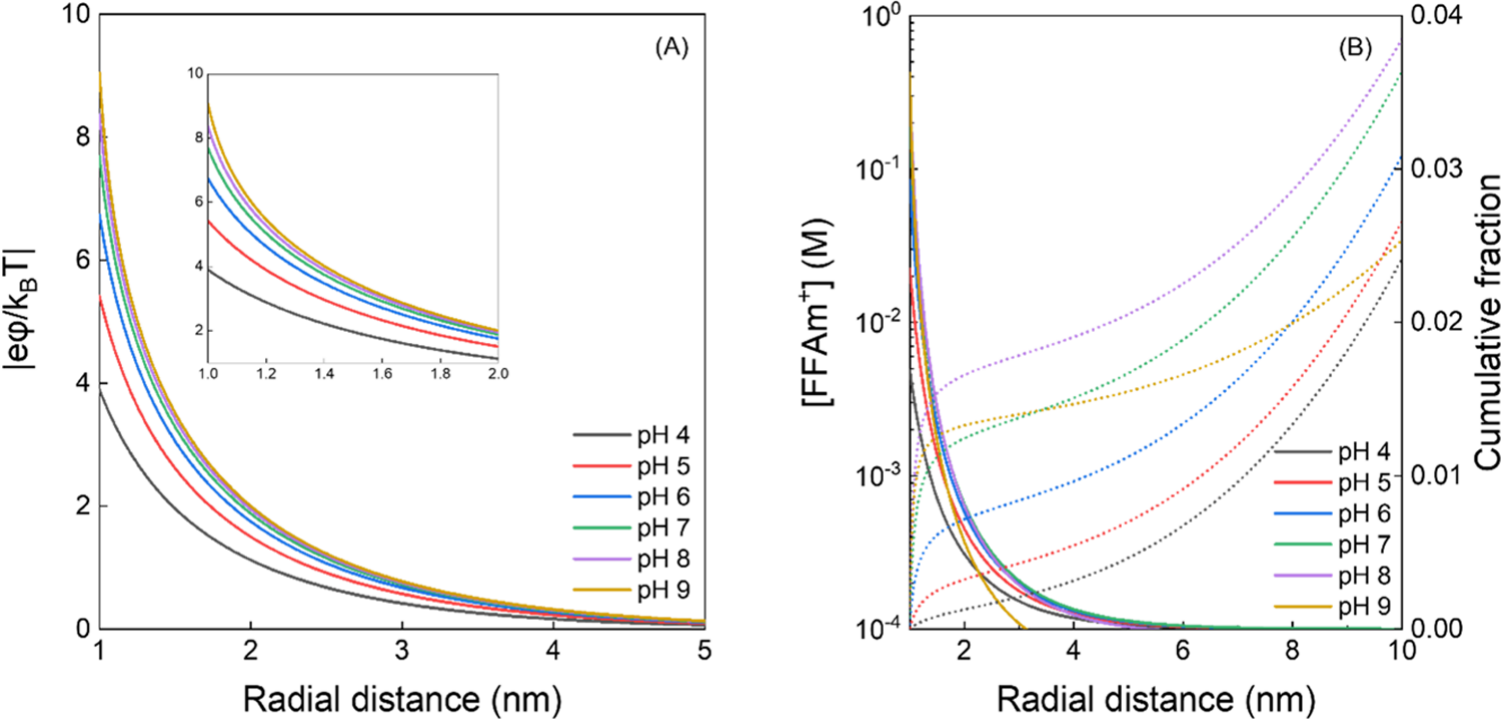
Characterization of the electrical potential and accumulation of cationic furfuryl amine for Suwannee River humic acid (SRHA) as a function of pH. (A) Dimensionless potential of SRHA (20 mg/L) as a function of distance at different pH values at 298 K; (B) concentration of cationic FFAm as a function of distance at different pH (primary axis); cumulative fraction of cationic FFAm as a function of distance at different pH (secondary axis). Concentration of cationic FFAm drops to 91 μM at pH 8 and 50 μM at pH 9 due to ion speciation. K^+^ dissociated from phosphate buffer with a concentration of 15 mM was considered as competing ions when determining the surface potential for solving for Poisson–Boltzmann equation. Data were acquired by numerically solving eq 1 via the successive approximation method. Calculations are described in Text S7.

Charge density calculations indicate that the surface potential of SRHA decreased from φ_s_ = −99.9 mV at pH 4 to −214.7 mV at pH 8. φ_s_ values for SRHA (Table S10) are appreciably higher than the potential at which the Debye–Hückel approximation breaks down, which justifies a numerical solution.^43^ With the ionic strength and competition from K^+^ cations from the potassium phosphate buffer considered, Figure 4 shows the numerically determined φ as a function of distance from the center of an SRHA molecule (*R* = 1 nm). φ decreased rapidly from 1 to 5 nm and declined to nearly zero by ∼10 nm. The resulting [FFAm^+^] distribution calculated from the Boltzmann equation varied greatly as a function of pH. For example, the calculated surface concentration of FFAm^+^ at pH 8 (0.39 M) was 79.6-fold higher than that at pH 4 (0.0049 M) due to SRHA’s more negative surface potential at pH 8. The spatial distribution of [FFAm^+^] determined above permits calculation of the cumulative fraction of FFAm^+^ residing within a certain distance (Figure 4B). For example, the fraction of FFAm^+^ residing within 10 nm of SRHA molecules relative to the total number of FFAm is 0.024 at pH 4 and increases to 0.038 at pH 8. At pH 9, the fraction was only 0.025 because approximately half of the total FFAm exists as a neutral species.

#### Solving for [^1^O_2_]_corona_

As shown in eq 4, FFAm experienced an apparent ^1^O_2_ concentration that depends on its distribution between phases and the volume averaged ^1^O_2_ concentration present in each phase.^3^ To solve for [^1^O_2_]_corona_, it is first necessary to determine the boundary of the corona region to obtain the fraction of FFAm^+^ within it. We approached this problem by relating the EF in terms of eq 3 (EF = [^1^O_2_]_app_/[^1^O_2_]_aq_) to the solution of the Boltzmann equation in spherical geometry, which ultimately leads to the corona length as a variable in response to the enhancement factor observed in photolysis experiments. As illustrated in Text S8, the length of corona region for SRHA at pH 8 was calculated as 18.80 nm. The cumulative fraction of FFAm^+^ located within 18.80 nm was determined to be 0.17. By applying this value to eq 4, the average concentration of ^1^O_2_ in the region determined by the corona length, [^1^O_2_]_corona_, was calculated to be 3.71 pM, which is 5.93-fold greater than the [^1^O_2_]_aq_ of 0.62 pM measured by FFA. At pH 4, the weaker electrostatic effect results in a lower accumulated fraction of FFAm^+^. This fraction was similar to neutral FFA (Figure S8), especially in the near-SRHA region. Thus, the modeling calculations are consistent with the reactivity enhancement observed from photolysis experiments. For example, EF ≈ 1 for SRHA at pH 4, which indicates that FFAm and FFA experienced largely the same [^1^O_2_]_app_ as a collective result of the similar spatial distribution of the probes. At pH 8, the magnitude of the negative surface potential of SRHA increased by a factor of 2 (−214.7 mV) based on modeling calculations. Accordingly, the electrostatic attraction of FFAm^+^ to SRHA was exponentially enhanced, resulting in a greater accumulated fraction of FFAm in the near-surface region and a larger *k*_obs_.

#### ^1^O_2_ Spatial Distribution in SRHA Corona Region

**Figure 5:**
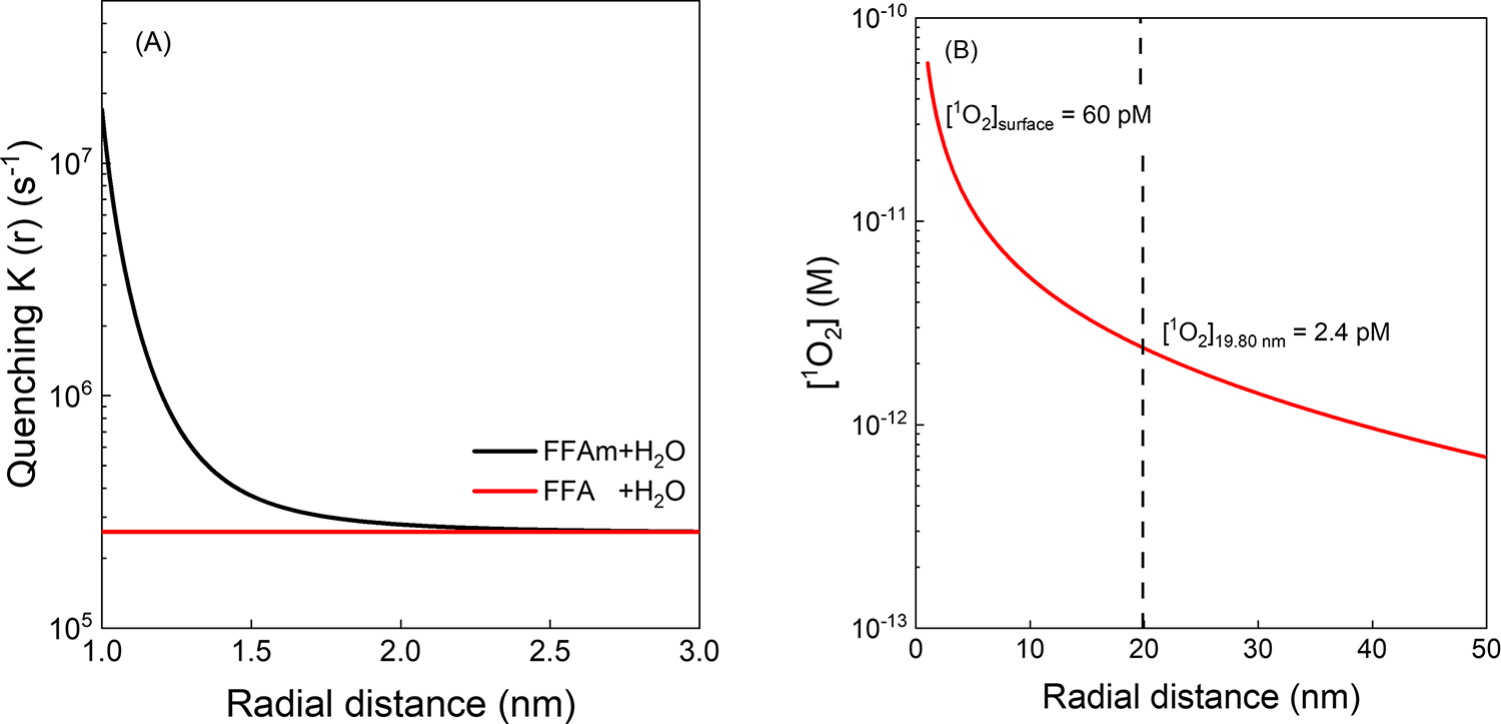
(A) Total quenching of ^1^O_2_ was calculated for FFAm and FFA-added solutions at a concentration of 100 μM in a 20 mg/L SRHA-containing solution at pH 8. (B) Spatial distribution of ^1^O_2_ in SRHA (20 mg/L) with a 1 nm radius at pH 8. SRHA molecular weight was estimated at 2329 Da from size exclusion chromatography measurements. The data were obtained by solving eq 6, and the mathematical steps are described in Text S9.

Following the Poisson–Boltzmann modeling, we sought to describe the spatial distribution of [^1^O_2_] in the DOM corona. As ^1^O_2_ diffuses away from the DOM surface, the major loss processes are physical quenching by H_2_O solvent and reactive quenching by FFA and FFAm. The resulting quenching kinetics can be expressed as (eq 5)
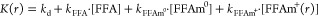
5where *K*(*r*) denotes the quenching to ^1^O_2_ as a function of radial distance, *k*_d_ = 2.5 × 10^5^ s^–1^ as solvent quenching rate constant, and *k*_FFA_ = 1.0 × 10^8^ M^–1^ s^–1^.^29^*k*_FFAm_^0^ = 2.07 × 10^8^ M^–1^ s^–1^ is the bimolecular rate constant of neutral FFAm, while *k*_FFAm_^+^ = 4.32 × 10^7^ M^–1^ s^–1^ is the bimolecular rate constant of FFAm^+^. FFA is evenly distributed in solution with a homogeneous concentration of 100 μM, whereas the distance dependence of [FFAm^+^] is derived from the Poisson–Boltzmann equation (eq 1). The results revealed that FFAm^+^ dominated the quenching term only near the SRHA surface (∼1 to 4 nm, depending on the pH, as shown in Figure 5A), while solvent quenching became more important with increasing distance. For example, at the SRHA surface (pH 8), the quenching due to FFAm (1.71 × 10^7^ s^–1^) was much greater than the sum of quenching by FFA and water inactivation (∑ = 0.1 × 10^5^ s^–1^ + 2.5 × 10^5^ s^–1^). The mathematical modeling, based on the quenching analysis, yields quantitative results of the accumulation of FFAm in DOM surroundings. It also explains how this accumulation enables the capture of more ^1^O_2_ than FFA in the DOM near-surface region.

The concentration of ^1^O_2_ (denoted by *C*_*t*_) changes as a function of time due to the source and sink processes in the steady state, which can be expressed by reaction–diffusion kinetic model. The quenching process (eq 5), as defined above in mathematical expression, has now been combined with the diffusion process to describe the distribution of ^1^O_2_.
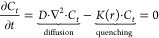
6where *D* = 2.3 × 10^–5^ cm^2^ s^–1^ is the diffusivity of O_2_ in water. In order to solve the equation and specify the behavior of ^1^O_2_ attenuation, two boundary conditions are required. One of these conditions is *C*_*t*_(*r* = ∞) = 0. The second boundary condition (Figure 5B), which applies [^1^O_2_]_corona_ = 3.71 pM, has been established to constrain the behavior of ^1^O_2_ attenuation from 1 to 19.8 nm radial distance (see Text S9).

By numerically solving the partial differential equation, we derived the spatial distribution profile of [^1^O_2_]. As shown in Figure 5B, at pH 8, the calculated [^1^O_2_] at the SRHA surface was 60 pM, which was attenuated to 2.4 pM at the corona boundary (19.80 nm). Based on these results, the [^1^O_2_]_surface_/[^1^O_2_]_aq_ ratio is 96 at pH 8. These values are similar to prior reports that determined 1–3 orders of magnitude higher [^1^O_2_] in DOM than in aqueous solution. Appiani et al.^20^ used Aldrich humic acid at a concentration of 10 mg/L and determined a [^1^O_2_] inside DOM phase as ∼31 pM, which is 63-fold greater than the aqueous phase measured by FFA. Grandbois et al. measured [^1^O_2_]_DOM_ to [^1^O_2_]_aq_ ratios for different DOM samples ranging from 100 to 1600, with the ratio for SRHA being 220.^9^ Using histidine, Chu et al.^11^ obtained a [^1^O_2_]_DOM_/[^1^O_2_]_aq_ ratio of 5500 ± 1000 for SRNOM, which is substantially higher than our [^1^O_2_]_surface_/[^1^O_2_]_aq_ value measured at pH 8 for SRHA. One possible explanation for this discrepancy is that Chu et al.^11^ considered only histidine sorption when calculating its fractional distribution between phases. If outer sphere electrostatic interactions enhanced the amount of histidine in the DOM vicinity, this would result in a higher fraction of histidine in close spatial proximity to DOM and consequently a lower calculated [^1^O_2_]_surface_/[^1^O_2_]_aq_.

There are several explanations possible for the differences in [^1^O_2_] spatial distribution at varying pH. The measured [^1^O_2_] depends on formation and scavenging rates, which in turn could vary with pH due to changes in apparent quantum yield, absorbance, and ^1^O_2_ reaction rate constant with DOM.^7^ Changes in DOM geometry as a function of pH could also impact the measured [^1^O_2_]. Studies have indicated a more rigid and compact DOM structure under acidic environment.^40,44,45^ A compact structure would have a lower surface-area-to-volume ratio, leading to more DOM-phase quenching and a less ^1^O_2_ flux to the DOM surface. In contrast, ionization of carboxyl and phenolic moieties with increasing pH would result in charge repulsion and a high surface-area-to-volume ratio, thereby increasing the ^1^O_2_ flux into the corona phase.

## Environmental Significance

It is expected that enhanced [^1^O_2_] in the near-DOM phase will increase rates of indirect photolysis of pollutants that partition to DOM. However, current photochemical models utilize bulk, aqueous-phase concentrations of [^1^O_2_].^46,47^ These models could potentially be improved by incorporating higher [^1^O_2_]_app_ for cationic and hydrophobic compounds. Enhanced [^1^O_2_] in the DOM vicinity may also lend support for the importance of this reactive species in the generation of photo-oxidized DOM, in particular, carboxylic-rich alicyclic molecules (CRAM).^5^

Results from this study provide support that [^1^O_2_] concentrations in the DOM vicinity are higher than those in the bulk aqueous phase, consistent with prior reports.^4,8,9,11,12^ The novel contributions from this study are (i) the use of the structurally related probe pair of furfuryl amine and furfuryl alcohol, (ii) application of the probe pair to a larger collection of DOM isolates than has been explored previously, and (iii) the combination of electrostatic modeling with the three-phase distribution model. We expect that future studies can take advantage of the probe pair approach and electrostatic models to measure [^1^O_2_]_corona_/[^1^O_2_]_aq_ in DOM from diverse biogeochemical contexts.

Although our study examined a large suite of DOM isolates from the International Humic Substances Society, application of the electrostatic model was limited to SRHA because the radius for this isolate is well constrained in the literature.^9^ Better measurements of DOM size would allow application to other samples and enhance confidence in the modeling results.

Several future experiments could help to more fully elucidate the structural features of DOM governing the extent of [^1^O_2_] microheterogeneity. The sensitivity of the probe pair approach could be improved by functionalizing the amine moiety to enhance hydrophobicity and create a permanent positive charge (e.g., *N*-pyridinium^48^). Other future experiments include using the probe pair to examine the impact of DOM molecular size (e.g., through ultrafiltration) and structure (e.g., through reduction of carbonyl groups with sodium borohydride) on [^1^O_2_]_app_. Such results would help to unravel whether the inverse dependence of ^1^O_2_ quantum yields on DOM size is governed by DOM microheterogeneity or size-driven changes in the rate of nonradiative deactivation of singlet excited state DOM.

## Supporting Information Available

Analytical methods, reaction kinetcics, calculation of DOM radius, and derivation of nonlinear Poisson–Boltzmann equation (Text); reaction rate constants, slope ratios, titration data, and surface potential (Tables); and absolute spectral irradiance of UV lamps, control experiment, and enhancement factor plotted against the charge density for a variety of DOMs (Figures) (PDF)
