# Supplementary material for: Evaluating the Microheterogeneous Distribution of Photochemically Generated Singlet Oxygen Using Furfuryl Amine
Source: Environ Sci Technol. 2023 May 2;57(19):7568–77. doi: 10.1021/acs.est.3c01726 (PMC10853930; doi:10.1021/acs.est.3c01726)
Supplement: Supplementary file 1 — es3c01726_si_001.pdf [file es3c01726_si_001.pdf]

Supporting Information for

Evaluating the Microheterogeneous Distribution of Photochemically Generated Singlet Oxygen using Furfuryl Amine

Kai Cheng[1], Lizhong Zhang[2], and Garrett McKay[1,*]

1. Zachry Department of Civil & Environmental Engineering, Texas A&M University, College Station, TX 77845
2. Department of Physics, University of California, Santa Barbara, Santa Barbara, CA 93106

*Corresponding author

gmckay@tamu.edu; (979) 458-6540

Number of Pages: 30

Number of Text Sections: 9

Number of Tables: 10

Number of Figures: 8

Table of Contents

Text S1. Analytical methods. 4

Text S2. Reaction kinetics in perinaphthenone-sensitized system 4

Text S3. Reaction kinetics in DOM-sensitized system. 6

Text S4. Evaluation of triplet-state DOM on the phototransformation of FFA and FFAm. 7

Test S5. Calculation of DOM radius 8

Text S6. Calculation of surface potential for DOM molecule. 8

Text S7. Derivation of non-linear Poisson-Boltzmann equation 9

Text S8. Mathematical approach for the determination of boundary distance of corona region.. 11

Text S9. Spatial distribution of $^1O_2$ derived from quenching analysis in diffusion process. 13

Table S1. List of chemicals used in this study. 16

Table S2. Control experiments free of sensitizers to evaluate the direct photolysis of the probes 16

Table S3. Reaction rate constants of FFA and FFAm at different pH obtained from homogeneous system with perinaphthenone as the sensitizer at 10 μM 17

Table S4. The slope ratios of kobsFFAm/ kobsFFA in perinaphthenone-sensitized system with increasing concentration of sodium formate at pH 8 17

Table S5. The evaluation of kinetic solvent isotope effect in D_2O. 17

Table S6. The slope ratios of kobsFFAm/ kobsFFA in DOM system. 18

Table S7. First-order rate constants for FFA and FFAm at pH 8 in DOM-sensitized solution at varying ionic strengths (IS). 19

Table S8. Titration data of DOM isolates referenced from IHSS 20

Table S9. Overall charge density (eq/mol) of DOM isolate obtained from modified Henderson-Hasselbalch model. 20

Table S10. Surface potential (mV) of SRHA isolate determined from charge density. 21

Figure S1. Absolute spectral irradiance of UV lamps with 365 nm wavelength, and the absorbance of perinaphthenone at the concentration of 10 μM and SRHA at 20 mg/L 22

Figure S2. Ratio of observed first-order rate constants of FFAm over FFA in SRHA with the addition of methanol as the quencher to •OH 22

Figure S3. Control experiment to evaluate the participation of triplet-state sensitizer in the photodegradation of FFA and FFAm. 23

Figure S4 Experimentally measured phototransformation rate constants of krxnFFAm plotted against solution pH in homogeneous system 24

Figure S5. The The observed first order rate constants of FFA and FFAm in perinaphthenone-sensitized system at varying pHs 25

Figure S6. The observed first order rate constants of FFA and FFAm measured from different DOMs sensitized solutions at varying pHs 27

Figure S7. Enhancement factor plotted against the charge density for a variety of DOMs 28
Figure S8. Cumulative fraction of FFAm (pH 4 and 8) and FFA in the vicinity of all SRHA molecules 29
References.. 30

Text S1. Analytical methods.

FFA and FFAm were used as probes to quantify the concentration of singlet oxygen during photolysis. Concentrations of FFA and FFAm were measured using ultra high-pressure liquid chromatography (UPLC) coupled with diode array detector and C16 column (Ascentis Express 90 Å RP-Amide 15 cm × 4.6 mm, 5 μm, Supelco). The injection volume was 50 μL, and the thermostatic column compartment was set to 30 °C. In homogenous system, the mobile phase was a mixture of 0.025 M ammonium acetate (A) at pH 9.5, and acetonitrile (B). The flow rate was 1 mL/min with the following solvent gradient: 0-3 min: 70% A and 30% B; 3-4 min: 70% A decreased to 50% A, 30% B increased to 50% B; 4-7 min: 50% A and 50% B; 7-8 min: 50% A increased to 70% A, 50% B decreased to 30% B; 8-10 min: 70% A and 30% B. Total elution time was 10 min with FFA eluted at 1.8 min, FFAm eluted at 2.4 min, and perinaphthenone eluted at 6.1 min. In microheterogeneous system, the mobile phase was an isocratic mixture of 0.025 M ammonium acetate (A) at pH 9.5 and acetonitrile (B) with 75% A and 25% B. Total elution time was 3.5 min with a flow rate at 1 mL/min. FFA had a retention time of 1.9 min, and FFAm had a retention time of 2.6 min. The UV adsorption data at 219 nm were collected.

Text S2. Reaction kinetics in perinaphthenone-sensitized system.

$^1O_2$ is formed through the energy transfer of triplet excited state DOM to molecular oxygen. In previous studies, FFA has been well-characterized for measuring the $^1O_2$ in aqueous solution.[1,2] By employing FFA as the reference, the reaction rate constant between FFAm and $^1O_2$ can be determined. Prepared in identical solution conditions and irradiated under the identical UV lamps simultaneously, FFA or FFAm (100 μM each, spiked in separate experimental tubes) were assumed to be experiencing the same amount of $^1O_2$ in perinaphthenone-sensitized system.

$$FFA + {}^1O_2 \xrightarrow{k_{rxn}^{FFA}} P_{FFA} \quad \text{(S2.1)}$$

$$FFAm + {}^1O_2 \xrightarrow{k_{rxn}^{FFAm}} P_{FFAm} \quad (S2.2)$$

The notations of k_{rxn}^{FFA} and k_{rxn}^{FFAm} denote the bimolecular rate constant of $^1O_2$ with FFA and FFAm. The rate law expressions of above reactions are described as:

$$-\frac{d[FFA]}{dt} = k_{rxn}^{FFA} \cdot [{}^1O_2]_{aq} \cdot [FFA] \quad (S2.3)$$

$$-\frac{d[FFAm]}{dt} = k_{rxn}^{FFAm} \cdot [{}^1O_2]_{aq} \cdot [FFAm] \quad (S2.4)$$

Given that probes are exposed to $[^1O_2]_{aq}$ in steady state ($[^1O_2]_{ss}$), the phototransformation rates of FFA and FFAm can be expressed in *pseudo-first-order* reaction kinetics.

$$-\frac{d[FFA]}{dt} = k_{rxn}^{FFA} \cdot [{}^1O_2]_{ss} \cdot [FFA] = k_{obs}^{FFA} \cdot [FFA] \quad (S2.5)$$

$$-\frac{d[FFAm]}{dt} = k_{rxn}^{FFAm} \cdot [{}^1O_2]_{ss} \cdot [FFAm] = k_{obs}^{FFAm} \cdot [FFAm] \quad (S2.6)$$

The concentrations of FFA and FFAm were monitored during photolysis and their transformation rates were fitted to a linear regression, yielding an observed first-order rate constant (k_{obs}). FFA is a well-characterized probe compound with a second-order rate constant at k_{rxn}^{FFA} =1.0 × 10^8 M^{-1}s^{-1}.[1] Therefore, $[^1O_2]_{ss}$ can be determined by

$$[{}^1O_2]_{ss}^{FFA} = \frac{k_{obs}^{FFA}}{k_{rxn}^{FFA}} \quad (S2.7)$$

As FFAm experiences the same $[^1O_2]_{ss}$ as FFA ($k_{rxn}^{probe}[probe] \ll 2.5 \times 10^5 s^{-1}$), the bimolecular rate constant of FFAm can be determined accordingly by

$$k_{rxn}^{FFAm} = \frac{k_{obs}^{FFAm}}{[{}^1O_2]_{ss}^{FFA}} = \frac{k_{obs}^{FFAm} \times k_{rxn}^{FFA}}{k_{obs}^{FFA}} \quad (S2.8)$$

k_{rxn}^{FFAm} is the apparent rate constant of FFAm as a function of pH. The speciation of FFAm at different pH results in different fractions (α) of cationic and neutral FFAm. Therefore, the apparent rate constant of FFA can be expressed by cationic FFAm (k_{rxn}^{FFAm+}) and neutral FFAm (k_{rxn}^{FFAm0}) as a function of speciation:

$$k_{rxn}^{FFAm} = \alpha_{FFAm^+} \cdot k_{rxn}^{FFAm^+} + \alpha_{FFAm^0} \cdot k_{rxn}^{FFAm^0} \quad \text{(S2.9)}$$

α denotes the fraction of cationic or neutral from of FFAm. Given the pH and pK_a of an organic acid or base, we can determine the fraction of the compound being ionized. S2.9 can be rewritten as,

$$k_{rxn}^{FFAm} = \frac{1}{1+10^{(pH-pKa)}} \cdot k_{rxn}^{FFAm^+} + \frac{10^{(pH-pKa)}}{1+10^{(pH-pKa)}} \cdot k_{rxn}^{FFAm^0} \quad \text{(S2.10)}$$

Fitting equation S2.10 to the experimental data yields a pK_a 9.01 ± 0.07 for FFAm, which is in good agreement with the literature value.[3] The bimolecular reaction rate constant of $^1O_2$ with cationic FFAm (k_{rxn}^{FFAm+}) is 4.32×10^7 M^{-1} s^{-1}, and the value of neutral FFAm (k_{rxn}^{FFAm0}) is 2.07×10^8 M^{-1} s^{-1}.

Text S3. Reaction kinetics in DOM-sensitized system.

The DOM system is referred to as microheterogeneous system, in which $^1O_2$ is nonuniformly distributed, with higher concentrations inside and near DOM relative to bulk, aqueous solution. Under this condition, the apparent concentrations of $^1O_2$ experienced by FFA and FFAm are different.

$$k_{obs}^{FFA} = k_{rxn}^{FFA} \cdot [^1O_2]_{app}^{FFA} \quad \text{(S3.1)}$$

$$k_{obs}^{FFAm} = k_{rxn}^{FFAm} \cdot [^1O_2]_{app}^{FFAm} \quad \text{(S3.2)}$$

The ratio of $k_{obs}^{FFAm}/k_{obs}^{FFA}$ between the DOM and the perinaphthenone system is defined as enhancement factor (*EF*) in the main content as eq.3. Applying equation S3.1 and S3.2 to expand the first-order rate constant of FFAm and FFA, the bimolecular rate constants get canceled out in the equation S3.3. Therefore, EF represents the differences in [$^1O_2$] experienced by the two probe compounds in DOM solution.

$$EF = \frac{(k_{obs}^{FFAm}/k_{obs}^{FFA})_{DOM}}{(k_{obs}^{FFAm}/k_{obs}^{FFA})_{PN}} = \frac{\left(\frac{k_{rxn}^{FFAm}\cdot[^1O_2]_{app}^{FFAm}}{k_{rxn}^{FFA}\cdot[^1O_2]_{app}^{FFA}}\right)_{DOM}}{\left(\frac{k_{rxn}^{FFAm}\cdot[^1O_2]_{ss}}{k_{rxn}^{FFA}\cdot[^1O_2]_{ss}}\right)_{PN}} = \left(\frac{[^1O_2]_{app}^{FFAm}}{[^1O_2]_{app}^{FFA}}\right)_{DOM} \quad \text{(S3.3)}$$

Text S4. Evaluation of triplet-state DOM on the phototransformation of FFA and FFAm.

To evaluate the participation of triplet-state sensitizer in the phototransformation of FFA and FFAm, control experiments were conducted by purging nitrogen gas to remove oxygen before photolysis. Experimental solutions were prepared in the flasks buffered by phosphate with FFA and FFAm spiked at 500 μM. Perinaphthenone was added at a concentration of 10 μM, while SRHA was added at a concentration of 20 mg/L. The solutions were then transferred to glass tube sealed with gas-tight caps. Nitrogen gas was used to purge the solutions for 3 min. The purging of nitrogen gas did not exhibit any volatilization of FFA or FFAm. Glass tubes were subsequently subjected to UV irradiation at 365 nm. At every time point, one glass tube was taken out to measure the concentration of FFA and FFAm. The results, which can be found in Figure S3, showed that there was no participation of triplet-state sensitizer in the degradation of FFA and FFAm in the absence of oxygen.

It should be noted that we observed the loss of perinaphthenone through volatilization during the nitrogen gas purging process. Spectrophotometry measurements showed that the concentrations of perinaphthenone decreased to approximately 5 μM after purging ((ε_{365} = 1.02 ×

10^4 $M^{-1}cm^{-1}$). However, even at this reduced concentration, the high quantum yield of perinaphthenone (ϕ_{1O2} = 0.95)[4] was sufficient to deplete the remaining oxygen within 1 minute. As a result, the observed results were not affected, and we were able to confirm the absence of triplet state sensitizer involvement in the phototransformation of the probe pairs.

Test S5. Calculation of DOM radius.

DOM radius can be obtained from its density and molecular weight by the following equation.

$$R = \sqrt[3]{\frac{3}{4\pi} \cdot \frac{M_n}{\rho \cdot N_A}} \tag{S5.1}$$

As reported from previous research,[5, 6] humic materials usually have a density around 1.5 g/cm^3. With its molecular weight determined as 2329 Da, SRHA was calculated to have a radius of 0.85 nm in spherical geometry. This is the number if solvent is completely excluded from the structure. Therefore, we adopted *R* = 1 nm for SRHA to allow for solvent inclusion.[7]

Text S6. Calculation of surface potential for DOM molecule.

Equation to solve for the electrical potential of a point charge is given as:

$$V(r) = \frac{q}{4\pi\varepsilon r} \tag{S6.1}$$

q in the equation has a unit of coulomb. Charge density of DOM molecule in eq/mol needs to be converted to coulomb using electron equivalent. For example, SRHA molecule at pH 4 was calculated to possess a charge density of 5.44 eq/mol (See Table S9). First, convert charge density per mole of SRHA to charge density per SRHA molecule.

$$\frac{5.4\ eq}{mol} \times \frac{mol}{\#6.022 \times 10^{23} SRHA} = 9.0 \times 10^{-24} \frac{eq}{SRHA} \tag{S6.2}$$

Then convert the electron equivalent to coulomb.

$$\frac{9.0\times10^{-24}eq}{SRHA}\times\frac{\#6.022\times10^{23}electron}{eq}\times\frac{-1.6022\times10^{-19}C}{electron}=\frac{-8.6\times10^{-19}C}{SRHA} \quad \text{(S6.3)}$$

Then apply equation S6.1 to derive surface potential in mV. The radius of SRHA is used as 1 nm:

$$V(r)=\frac{q}{4\pi\varepsilon_0\varepsilon_w r}=\frac{-8.6\times10^{-19}C}{SRHA}\times\frac{1}{4\pi\times8.854\times10^{-12}\frac{C^2}{J.m}\times78.5\times1nm\times\frac{10^{-9}m}{nm}}$$

$$=-0.099\frac{J}{C}=-99mV \quad \text{(S6.4)}$$

Text S7. Derivation of non-linear Poisson-Boltzmann equation.

The following approach adopted the views from ion-impermeable model that treats humic substances as an impermeable sphere with charges localized on the surface. First define the total charge density of the solution as

$$\rho_e=\sum_i z^{(i)}en^{(i)} \quad \text{(S7.1)}$$

where i refers to the ion species, $z^{(i)}$ is the charge number of the ion, and e is the elementary charge. Since the solution is in steady state, we can assume that the number density of each ion species $n^{(i)}$ satisfies the Boltzmann distribution.

$$n^{(i)}=n_0^{(i)}\cdot exp\left(-\frac{z^{(i)}e\varphi}{k_BT}\right) \quad \text{(S7.2)}$$

where $n_0^{(i)}$ is the number density of an ion in the bulk solution free from the influence of the electric field. It is a constant and can be determined via electroneutrality and the ion number conservation. The electrical potential φ can be solved by Gauss's law, which introduces the sourced Poisson equation as

$$\nabla^2\varphi = -\frac{\rho_e}{\varepsilon} \tag{S7.3}$$

where constant ε is the absolute permittivity of the solution. Selecting the species with $i = 0$ as a central ion, we have the electroneutrality of the solution as

$$z^{(0)}e + \int_V \rho_e \, dV = 0 \tag{S7.4}$$

Note that the volume-averaged number density of each species $\langle n^{(i)} \rangle_V$ is measurable. The conservation of ion number is then given by

$$\delta_{0i} + \int_V n^{(i)} \, dV = \langle n^{(i)} \rangle_V \cdot \int_V dV \tag{S7.5}$$

where δ_{0i} is the Kronecker delta. With the above five equations, the system is closed and ready to be solved.

Assume the electrical potential is isotropic around the central ion in the spherical geometry, the established system can be solved as a function of spherical radius r about the central ion. The governing equations are summarized below in the sequence of Poisson-Boltzmann equation, ion number conservation and electroneutrality.

$$\frac{d^2\varphi}{dr^2} + \frac{2}{r}\frac{d\varphi}{dr} + \frac{1}{\varepsilon} \cdot \sum_i z^{(i)} e n_0^{(i)} \cdot exp\left(-\frac{z^{(i)} e\varphi}{k_B T}\right) = 0 \tag{S7.6a}$$

$$\frac{\delta_{0i}}{4\pi} + n_0^{(i)} \cdot \int_V exp\left(-\frac{z^{(i)} e\varphi}{k_B T}\right) \cdot r^2 dr = \langle n^{(i)} \rangle_V \cdot \int_V r^2 dr \tag{S7.6b}$$

$$\sum_i z^{(i)} \cdot \langle n^{(i)} \rangle_V = 0 \tag{S7.6c}$$

The key variable for calculating the Coulombic effect on the energy of a reaction is the electrostatic potential φ , which is obtained as the solution of a Poisson-Boltzmann equation. This equation assumes that φ is created by a central charge region of interest.

Text S8. Mathematical approach for the determination of boundary distance of corona region.

From the three-region model proposed by Latch and McNeil,[8] the bulk, aqueous phase $[^1O_2]_{aq}$ measured by the probe molecule (FFA) is simply the $[^1O_2]_{corona}$ weighted by the fraction of total aqueous phase that the corona occupies, which leads to the following equation by mass balance:

$$[\,^1O_2]_{corona} \cdot V_{corona} = [\,^1O_2]_{aq} \cdot V_{tot} \tag{S8.1}$$

The enhancement factor equation is defined in Text S3 by equation S3.3 as

$$EF = \frac{[\,^1O_2]_{app}}{[\,^1O_2]_{aq}} \tag{S8.2}$$

As FFAm exists in the corona phase and bulk aqueous phase, $[\,^1O_2]_{app}$ can be expressed as a function of FFAm partitioning as follows,

$$[\,^1O_2]_{app} = f_{corona} \cdot [\,^1O_2]_{corona} + f_{aq} \cdot [\,^1O_2]_{aq} \tag{S8.3}$$

Substituting equation S8.1 and S8.2 into S8.3 yields:

$$EF \cdot [\,^1O_2]_{aq} = f_{corona} \cdot \frac{[\,^1O_2]_{aq} \cdot V_{tot}}{V_{corona}} + f_{aq} \cdot [\,^1O_2]_{aq} \tag{S8.4}$$

The equation can be further simplified by dividing both side with $[\,^1O_2]_{aq}$:

$$EF = f_{corona} \cdot \frac{V_{tot}}{V_{corona}} + f_{aq} \tag{S8.5}$$

For a 1 L solution $V_{tot} = 1L$. Factoring out V_{corona} and moving it to the left hand of the equation yields:

$$V_{corona} = \frac{f_{corona}}{EF - f_{aq}} \tag{S8.6}$$

Assuming that the boundary length of corona region is distance *x* from the DOM radius *R*, V_{corona} can determined from:

$$V_{corona} = N_s \cdot (\frac{4}{3}\pi(R+x)^3 - \frac{4}{3}\pi R^3) \quad \text{(S8.7)}$$

where N_s is the number of DOM molecules, which can be determined by its concentration (20 mg/L used in our experiments), and its molecular weight measured from size exclusion chromatography measurement (values presented in Table S8). In equation S8.6, the fraction of $FFAm^+$ as a function of distance can be derived from Poisson-Boltzmann solution as described in Text S7, which is mathematically expressed as:

$$f_{corona} = \frac{N_S \cdot \int C_{FFAm^+}(x) \cdot 4\pi(R+x)^2 dx}{M_{FFAm}} \quad \text{(S8.8)}$$

where $C_{FFAm^+}(x)$ is the concentration of cationic FFAm as a function of distance and M_{FFAm} is the total mass of FFAm spiked in solution. As the fraction of FFAm partitioning in corona region and in bulk solution add up to one, we have the equation below:

$$f_{aq} = 1 - f_{corona} \quad \text{(S8.9)}$$

Substituting equation S8.7, S8.8, and S8.9 into equation S8.6 yields:

$$N_s \cdot (\frac{4}{3}\pi(R+x)^3 - \frac{4}{3}\pi R^3) = \frac{\frac{N_S \cdot \int C_{FFAm^+}(x) \cdot 4\pi(R+x)^2 dx}{M_{FFAm}}}{EF - \left(1 - \frac{N_S \cdot \int C_{FFAm^+}(x) \cdot 4\pi(R+x)^2 dx}{M_{FFAm}}\right)} \quad \text{(S8.10)}$$

Below we show an example of calculating the corona boundary by performing an iterative approach in Excel spreadsheet to solve for equation S8.10. The first two columns were the data obtained from solving non-linear Poisson-Boltzmann equation using Python. The second column indicated the number accumulation of cationic FFAm with distance dependence around one SRHA

molecule. By multiplying the number of SRHA molecules, the total fraction of FFAm as in $f_{corona}(R+x)$ and $f_{aq}(R+x)$ can thereby be determined. Then performing iterative approach to the left-hand side and right-hand side of equation to derive the $R+x$.

SRHA at pH 8									
Exported data from P-B solution		Data processing					Solve equation S8.10		
Radius (nm)	Accumulated [FFAm+] (M)/SRHA molecule	Number of FFAm+/SRHA molecule	Number of FFAm+ of SRHA considered	Accumu FFAm fraction at each distance	Frac_aqueous		RHS	LHS	RHS-LHS
1	2.80E-26	1.68E-02	8.71E+16	0.14%	99.86%		0.00168	0.0000	0.0017
1.00624	5.18E-26	3.12E-02	1.61E+17	0.27%	99.73%		0.00311	0.0000	0.0031
1.01252	7.24E-26	4.36E-02	2.25E+17	0.37%	99.63%		0.00433	0.0000	0.0043
1.01884	9.03E-26	5.44E-02	2.81E+17	0.47%	99.53%		0.00540	0.0000	0.0054
1.0252	1.06E-25	6.38E-02	3.30E+17	0.55%	99.45%		0.00633	0.0000	0.0063
1.03159	1.20E-25	7.22E-02	3.73E+17	0.62%	99.38%		0.00716	0.0000	0.0072
1.03803	1.32E-25	7.97E-02	4.12E+17	0.68%	99.32%		0.00789	0.0000	0.0079
1.04451	1.43E-25	8.64E-02	4.46E+17	0.74%	99.26%		0.00855	0.0000	0.0085
1.05103	1.53E-25	9.24E-02	4.78E+17	0.79%	99.21%		0.00914	0.0000	0.0091
1.05758	1.63E-25	9.79E-02	5.06E+17	0.84%	99.16%		0.00968	0.0000	0.0097
1.06418	1.71E-25	1.03E-01	5.32E+17	0.88%	99.12%		0.01016	0.0000	0.0102
1.07082	1.78E-25	1.07E-01	5.55E+17	0.92%	99.08%		0.01061	0.0000	0.0106
1.07751	1.85E-25	1.12E-01	5.77E+17	0.96%	99.04%		0.01102	0.0000	0.0110
1.08423	1.92E-25	1.15E-01	5.97E+17	0.99%	99.01%		0.01139	0.0000	0.0114
1.091	1.98E-25	1.19E-01	6.15E+17	1.02%	98.98%		0.01174	0.0000	0.0117
1.0978	2.03E-25	1.22E-01	6.32E+17	1.05%	98.95%		0.01206	0.0000	0.0121
1.10466	2.08E-25	1.25E-01	6.48E+17	1.08%	98.92%		0.01236	0.0000	0.0124
1.11155	2.13E-25	1.28E-01	6.63E+17	1.10%	98.90%		0.01264	0.0000	0.0126
1.11848	2.17E-25	1.31E-01	6.76E+17	1.12%	98.88%		0.01289	0.0000	0.0129
1.12546	2.21E-25	1.33E-01	6.89E+17	1.14%	98.86%		0.01314	0.0000	0.0131
1.13249	2.25E-25	1.36E-01	7.01E+17	1.16%	98.84%		0.01336	0.0000	0.0134
1.13955	2.29E-25	1.38E-01	7.13E+17	1.18%	98.82%		0.01358	0.0000	0.0136
… …									
19.8063	3.37E-24	2.03E+00	1.05E+19	17.44%	82.56%		0.16859	0.1681	0.0004
19.9299	3.43E-24	2.06E+00	1.07E+19	17.73%	82.27%		0.17095	0.1713	-0.0004
20.0542	3.49E-24	2.10E+00	1.09E+19	18.03%	81.97%		0.17333	0.1745	-0.0012

Avagadro	6.02E+23	
[SRHA]	8.58738E-06	M
Tot #_SRHA	5.1696E+18	#
[FFAm]	0.0001	M
Tot #_FFAm	6.02E+19	

SRHA photochemical reaction		
pH	Time (h)	k_obs (h^-1)
7.979	FFA	0.225
	FFAM	0.242
k_rxn_FFA	100000000	M-1.s-1
k_rxn_FFAm	57800000	M-1.s-1
EF	1.86	
[1O2]app	1.16319E-12	M
	1.163189549	pM
[1O2]aq	6.24922E-13	M
	0.624922298	pM
[1O2]corona	3.7115	pM
Corona/aq	5.939	folds

Text S9. Spatial distribution of $^1O_2$ derived from quenching analysis in diffusion process.

Treating humic acid molecules as spherical microregions leads to three regions of different $^1O_2$ abundance: the interior of the sphere where the production occurs with the highest $^1O_2$ concentration, the aqueous corona region surrounding the sphere that receives the flux of $^1O_2$, and the aqueous phase that lies out the diffusion region. In the ion-impermeable model, averaged concentration of $^1O_2$ in corona region is reported as a result of applying Poisson-Boltzmann equation and corona boundary length calculation. In the process of diffusion, the main loss processes include quenching by water, and quenchers of FFA, and FFAm. Thus, the quenching kinetics can be expressed as:

$$K(r) = k_d + k_{FFA} \cdot [FFA] + k_{FFAm^0}[FFAm^0] + k_{FFAm^+}[FFAm^+(r)] \qquad \text{(S9.1)}$$

where K(r) denotes the quenching to $^1O_2$ as a result of solvent and probes. k_d =2.5 × 10^5 s^{-1} as solvent quenching rate constant, k_{FFA} =1.0 × 10^8 M^{-1} s^{-1}.[1] k_{FFAm^0} = 2.07 × 10^8 M^{-1} s^{-1} is the bimolecular rate constant of neutral FFAm while k_{FFAm^+} =4.32 × 10^7 M^{-1} s^{-1} is the bimolecular rate constant of $FFAm^+$. FFA is evenly distributed in solution with a homogenoues concentration at 100 μM whereas FFAm has a concentration with distance dependence that is derived from Piosson-Boltzmann equation. As DOM contributed to insignificant amount of quenching to $^1O_2$ loss, the concentration of $^1O_2$ (denoted by C_t) can be determined by

$$\frac{\partial C_t}{\partial t} = \underbrace{D \cdot \nabla^2 \cdot C_t}_{diffusion} - \underbrace{K(r) \cdot C_t}_{quenching} \quad \text{(S9.2)}$$

where $D = 2.3 \times 10^{-5} cm^2/s$ as diffusivity of O_2 in water. Assume the solution is in steady state that the concentration of $^1O_2$ remains unchanged as the same amount of flux of $^1O_2$ is produced and diffuses out of DOM molecule,

$$\frac{\partial C_t}{\partial t} = 0 = D \cdot \nabla^2 \cdot C - K(r) \cdot C_t \quad \text{(S9.3)}$$

Expand the LaPlace in spherical geometry, the equation becomes

$$\frac{D}{r^2} \cdot \frac{\partial}{r} (r^2 \cdot \frac{\partial C_t}{\partial r}) - K(r) \cdot C_t = 0 \quad \text{(S9.4)}$$

Divide both sides by D and multiply the items in parenthesis,

$$\frac{\partial^2 C_t}{\partial r^2} + \frac{2}{r} \cdot (\frac{\partial C_t}{\partial r}) - \frac{K(r)}{D} \cdot C_t = 0 \quad \text{(S9.5)}$$

Clean up the equation

$$\begin{cases} \frac{\partial C_t'}{\partial r} = \frac{K(r)}{D} \cdot C_t - \frac{2}{r} \cdot C_t' \\ \frac{\partial C_t}{\partial r} = C_t' \end{cases} \quad \text{(S9.6)}$$

Boundary conditions to solve for the partial differential equations are

$$\begin{cases} C_t(r = \infty) = 0 \\ \frac{\int_R^{r_{corona}} C_t \cdot 4\pi r^2 dr}{\frac{4}{3}\pi r_{corona}^3} = C_{avg} \end{cases} \tag{S9.7}$$

Where R is the radius of DOM molecule and the r_{corona} is the boundary distance determined from previous calculations in Text S8. In order to solve the equation and specify the behavior of $^1O_2$ attenuation, two boundary conditions are required. One of these conditions is $C_t(r = \infty) = 0$. The second boundary condition (Figure 5B), which applies $[^1O_2]_{corona}$, has been established to constrain the behavior of $^1O_2$ attenuation from DOM radius (R) to corona length (r_{corona}) that contains C_{avg} as the average concentration of $^1O_2$ in corona region determined by previous calculations using eq. 4 in main content.

Table S1. List of chemicals used in this study.

Chemical	Cas #	Purity	Supplier
Perinaphthenone	548-39-0	>97%	Aldrich
Furfuryl alcohol	RN-98-00-0	>98%	TCI America
Furfuryl amine	617-89-0	>99%	Aldrich
Dipotassium hydrogen phosphate	7758-11-4	98.0%	VWR Chemicals
Potassium dihydrogen phosphate	7778-77-0	>99.0%	VWR Chemicals
Ammonium acetate	631-61-8	>99.99%	Aldrich
Acetonitrile	75-05-8	>99.95%	VWR Chemicals
Potassium chloride	7447-40-7	>99.0%	Aldrich
Hydrochloric acid 2.0 N	7647-01-0	ACS grade	VWR Chemicals
Sodium hydroxide	1310-73-2	95.0-100%	VWR Chemicals
Methanol	67-56-1	100%	VWR Chemicals
Deuterium oxide	7789-20-0	>99.9%	Aldrich
Sodium formate	141-53-7	>99.0%	Aldrich
Deuterium chloride solution	7698-05-7	>99%	Aldrich
Sodium deuteroxide solution	14014-06-3	>99.5%	Aldrich

Table S2. Control experiments free of sensitizers to evaluate the direct photolysis of the probes.

Control	Probe	Concentration (μM)		Discrepancy (%)
		Time 0 h	Time 8 h	
1	FFA	493	491	0.41
	FFAm	486	475	2.26
2	FFA	511	510	0.20
	FFAm	496	494	0.40
3	FFA	528	510	3.41
	FFAm	480	477	0.63
4	FFA	493	491	0.41
	FFAm	525	511	2.67

Table S3. Reaction rate constants of FFA and FFAm at different pH obtained from homogeneous system with perinaphthenone as the sensitizer at 10 μM. Model-correction refers to bimolecular rate obtained by fitting the measured data to ion speciation equation (Text S2 and Figure S4).

pH	FFA		FFAm			Model-correction	
	k_{obs}^{FFA} (s^{-1})	StD	k_{obs}^{FFAm} (s^{-1})	StD	k_{obs}^{FFAm} ($M^{-1}s^{-1}$)	k_{obs}^{FFAm} ($M^{-1}s^{-1}$)	($k_{obs}^{FFAm}/k_{obs}^{FFA}$)
4	0.0101	0.0008	0.0043	0.0002	4.30E+07	4.32E+07	0.432
5	0.0103	0.0002	0.0044	0.0003	4.31E+07	4.32E+07	0.432
6	0.0126	0.0007	0.0057	0.0002	4.50E+07	4.34E+07	0.434
7	0.0104	0.0003	0.0055	0.0002	5.29E+07	4.48E+07	0.448
8	0.0122	0.0012	0.0074	0.0005	6.05E+07	5.78E+07	0.578
9	0.0102	0.0010	0.0126	0.0011	1.23E+08	1.24E+08	1.242
10	0.004	0.000	0.008	0.0003	1.92E+08	1.92E+08	1.918

Noted that StD represents standard deviation. Also, at pH 10, half of UV lamps were removed to avoid the depletion of probe compound. Intensity of light source was affected accordingly. k_{obs} therefore was inconsistent from other pHs.

Table S4. The slope ratios of $k_{obs}^{FFAm}/k_{obs}^{FFA}$ in perinaphthenone-sensitized system with increasing concentration of sodium formate at pH 8. FFAm and FFA were spiked at the concentration of 500 μM.

HCOONa (μM)	$k_{obs}^{FFAm}/k_{obs}^{FFA}$
0	0.845
50	0.890
100	0.858
200	0.816
500	0.846
1000	0.837

Table S5. The evaluation of kinetic solvent isotope effect in D_2O. SRHA at a concentration of 20 mg/L was used as sensitizer. Experiments were conducted in D_2O at identical condition at pD equal to 8.00 and 7.60. To exclude the influence of protons dissociated from phosphate buffer solution, KCl was used instead to achieve the ionic strength at the same level as buffer does.

FFA		k_D/k_H	FFAm		k_D/k_H
Solvent	k_{obs}^{FFA} (h^{-1})		Solvent	k_{obs}^{FFAm} (h^{-1})	
H_2O @ pH 8.0	0.225		H_2O @ pH 8.0	0.242	
D_2O @ pD 7.6	3.166	14.1	D_2O @ pD 7.6	1.160	4.8
D_2O @ pD 8.0	3.037	13.5	D_2O @ pD 8.0	1.125	4.6

Table S6. The slope ratios of k_{obs}^{FFAm}/ k_{obs}^{FFA} in DOM system. A variety of DOM isolates were used as sensitizer at 20 mg/L. FFA or FFAm was added with a concentration of 100 µM.

pH	SRHA		SRFA	
	k_{obs}^{FFAm}/ k_{obs}^{FFA}	StD	k_{obs}^{FFAm}/ k_{obs}^{FFA}	StD
4	0.404	0.020	0.388	0.009
5	0.419	0.012	0.395	0.013
6	0.446	0.018	0.436	0.017
7	0.580	0.008	0.576	0.019
8	1.076	0.028	0.911	0.037
9	1.673	0.021	1.795	0.012

pH	SRNOM		MRNOM	
	k_{obs}^{FFAm}/ k_{obs}^{FFA}	StD	k_{obs}^{FFAm}/ k_{obs}^{FFA}	StD
4	0.394	0.032	0.440	0.014
5	0.428	0.007	0.466	0.025
6	0.452	0.026	0.426	0.015
7	0.603	0.024	0.613	0.045
8	0.948	0.058	0.972	0.018
9	1.717	0.085	1.560	0.023

pH	PPHA		PPFA	
	k_{obs}^{FFAm}/ k_{obs}^{FFA}	StD	k_{obs}^{FFAm}/ k_{obs}^{FFA}	StD
4	0.502	0.021	0.387	0.008
5	0.439	0.019	0.423	0.008
6	0.495	0.034	0.421	0.007
7	0.627	0.023	0.558	0.021
8	0.998	0.013	0.812	0.033
9	1.519	0.077	1.352	0.028

pH	ESHA		ESFA	
	k_{obs}^{FFAm}/ k_{obs}^{FFA}	StD	k_{obs}^{FFAm}/ k_{obs}^{FFA}	StD
4	0.423	0.048	0.452	0.022
5	0.474	0.027	0.363	0.028
6	0.354	0.013	0.441	0.021
7	0.461	0.016	0.538	0.025
8	0.829	0.018	0.746	0.030
9	1.295	0.067	1.510	0.019

Table S7. First-order rate constants for FFA and FFAm at pH 8 in DOM-sensitized solution at varying ionic strengths (IS).

DOM	IS (mM)	k_{obs}^{FFA} (h^{-1})	k_{obs}^{FFAm}(h^{-1})	k_{obs}^{FFAm}/ k_{obs}^{FFA}
SRHA	20	0.225	0.242	1.076
	100	0.201	0.166	0.827
	200	0.201	0.145	0.720
SRFA	20	0.252	0.229	0.911
	100	0.223	0.182	0.817
	200	0.224	0.172	0.769
SRNOM	20	0.226	0.214	0.948
	100	0.229	0.169	0.741
	200	0.216	0.150	0.696
MRNOM	20	0.292	0.284	0.972
	100	0.250	0.187	0.749
	200	0.241	0.171	0.710
PPHA	20	0.687	0.686	0.998
	100	0.917	0.684	0.747
	200	1.017	0.809	0.795
PPFA	20	1.411	1.146	0.812
	100	1.305	1.123	0.861
	200	1.204	1.071	0.889
ESHA	20	1.517	1.258	0.829
	100	0.989	0.723	0.732
	200	0.899	0.657	0.730
ESFA	20	0.838	0.625	0.746
	100	0.735	0.575	0.782
	200	0.697	0.514	0.737

Table S8. Titration data of DOM isolates referenced from IHSS.

DOM	Carboxyl (meq/gC)	Phenolic (meq/gC)	Q_1	Log K_1	n_1	Q_2	Log K_2	n_2	C	Mn (Da)
SRHA	9.13	3.72	9.74	4.35	3.3	4.48	10.44	1.73	55%	2329
SRFA	11.17	2.84	11.66	3.76	3.24	2.05	9.84	1.45	53%	1436
MRNOM	11.21	2.47	11.2	4.16	3.44	1.6	9.99	1.03	51%	1611
SRNOM	12.43	0.83	12.51	3.47	2.69	0.91	10	1	50%	1748
PPHA	9.01	1.91	9.64	4.22	3.2	0.94	9.86	1	56%	2591
PPFA	13.34	2.32	14.22	3.99	3.33	0.76	9.57	1	51%	2310
ESHA	8.28	1.87	8.9	4.36	3.16	0.85	9.8	1	59%	2399
ESFA	13.24	2.27	14.12	3.67	3.62	0.74	9.53	1	52%	--

Note: Equation to solve for the charge density is shown by eq. 2 in the main content. The fitting parameters of the model were obtained by a nonlinear least-squares fit of the model to aggregated sets of replicate titration data. Q_1 and Q_2 are the maximum charge densities of the two classes of binding sites, $Log\ K_1$ and $Log\ K_2$ are the mean log K values for proton binding by the two classes of sites, and n_1 and n_2 are empirical parameters that control the width (in log K) of a class of proton binding sites. Molecular weight of ESFA was not measured.

Table S9. Overall charge density (eq/mol) of DOM isolate obtained from modified Henderson-Hasselbalch model.

pH	SRHA	SRFA	MRNOM	SRNOM	PPHA	PPFA	ESHA	ESFA
4	5.44	4.84	4.70	6.16	6.48	8.46	5.45	NA
5	7.58	6.31	6.32	7.93	8.97	11.26	7.71	NA
6	9.42	7.42	7.68	9.04	11.02	13.49	9.63	NA
7	10.76	8.13	8.64	9.61	12.40	14.99	10.95	NA
8	11.70	8.59	9.24	9.88	13.23	15.89	11.74	NA
9	12.65	9.04	9.69	10.05	13.81	16.53	12.30	NA

Note: Charge density is normally expressed in the unit of meq/gC, here in this table, the presented numbers were converted from meq/gC to eq/mol using the molecular weight determined from gel exclusion chromatography. Molecular weight of each DOM isolate was summarized in Table S8.

Table S10. Surface potential (mV) of SRHA isolate determined from charge density.

pH	SRHA (mV)
4	-99.9
5	-139.0
6	-173.0
7	-197.4
8	-214.7
9	-232.2

Note: The radius of one SRHA molecule was used as 1 nm. Charge density at each pH can be converted to surface potential by invoking equation for electrical potential due to a point charge. Detailed calculation is demonstrated in Text S6.

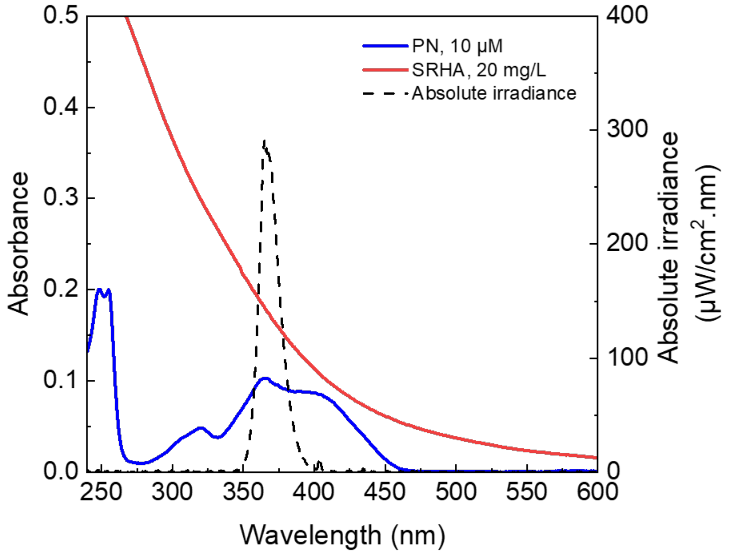

Figure S1. Absolute spectral irradiance of UV lamps with 365 nm wavelength, and the absorbance of perinaphthenone at the concentration of 10 μM and SRHA at 20 mg/L.

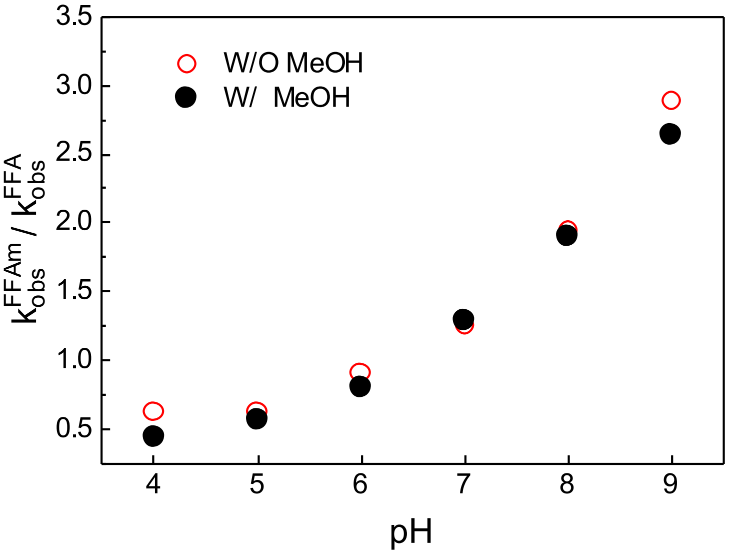

Figure S2. Ratio of observed first-order rate constants of FFAm over FFA in SRHA-sensitized microheterogeneous system with (black circle) and without (empty circle) the addition of methanol (100 mM) as the quencher to •OH.

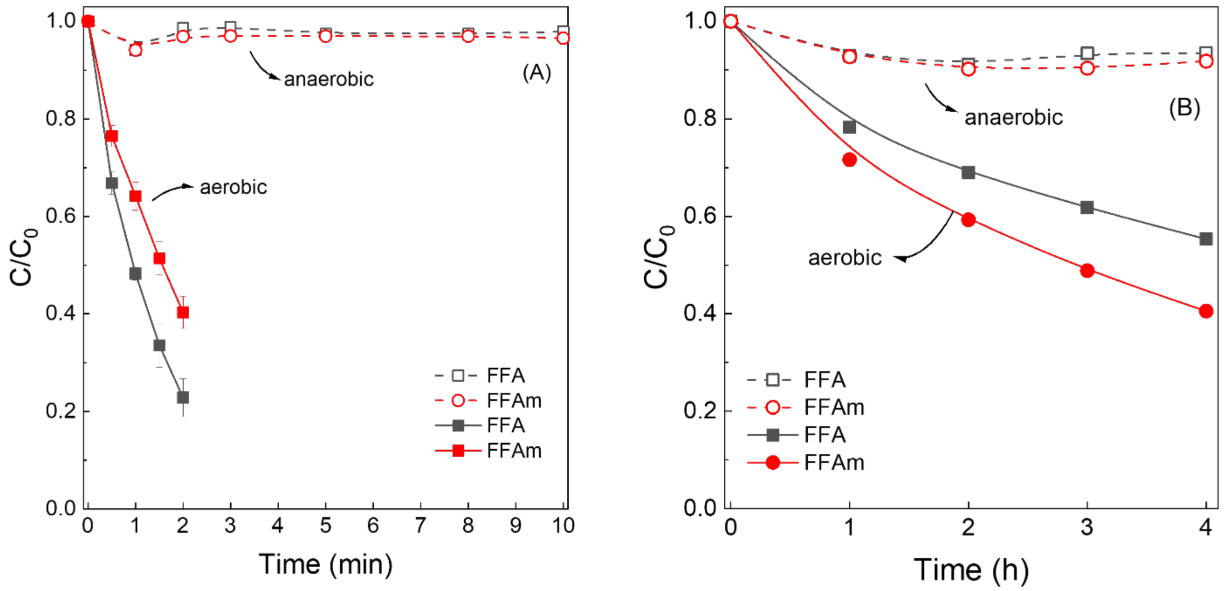

Figure S3. Control experiment to evaluate the participation of triplet-state sensitizer in the photodegradation of FFA and FFAm. Nitrogen was used to purge the solution to remove oxygen as much as possible before sealing the experimental tubes. Both probes were spiked with a concentration of 500 µM at pH 8 in aerobic and anaerobic environment. (A) Perinaphthenone (10 µM) was used as sensitizer. (B) SRHA (20 mg/L) was used as sensitizer. Solid squares denoted the degradation of FFA and FFAm in the presence of oxygen, while empty circles indicated the degradation of FFA and FFAm after nitrogen gas sparging.

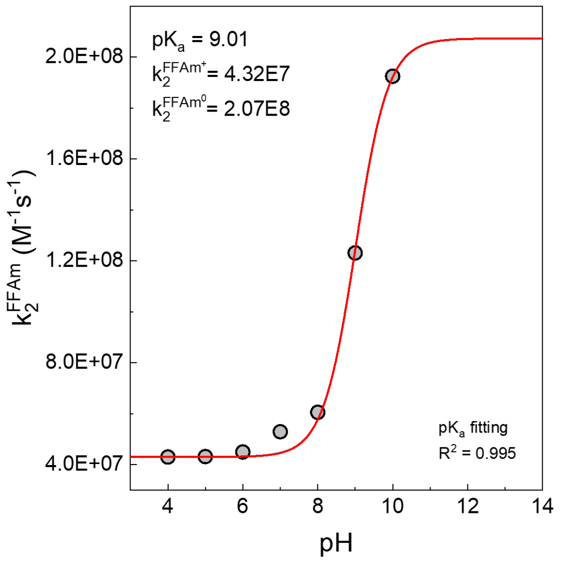

Figure S4 Experimentally measured phototransformation rate constants of k_{rxn}^{FFAm} plotted against solution pH in homogeneous system containing perinaphthenone at 10 µM. Data points are plotted at the average values from triplicates. The curve fit corresponds to FFAm speciation to determine the pKa and the rate constants of cationic FFAm and neutral FFAm. Fitting equation can be found from Text S2.

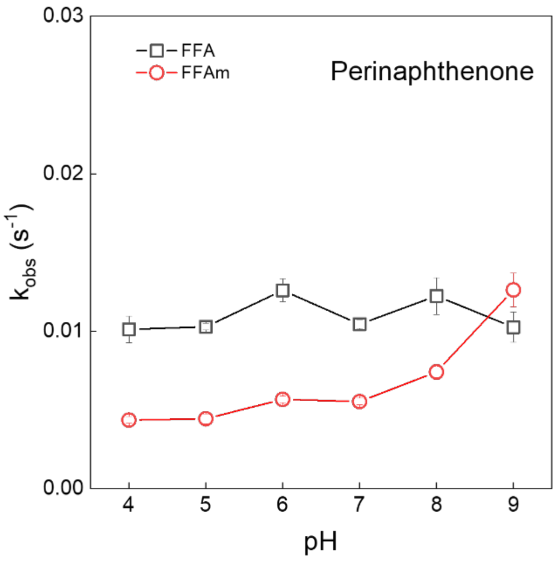

Figure S5. The observed first order rate constants of FFA and FFAm in perinaphthenone-sensitized system at varying pHs. Perinaphthenone had a concentration of 10 µM with FFA or FFAm (spiked in separate experimental tubes) added at 100 µM. Phosphate buffer (10 mM) was used to maintain the pH. Triplicates were conducted during the photolysis. The error bars indicated the standard deviation.

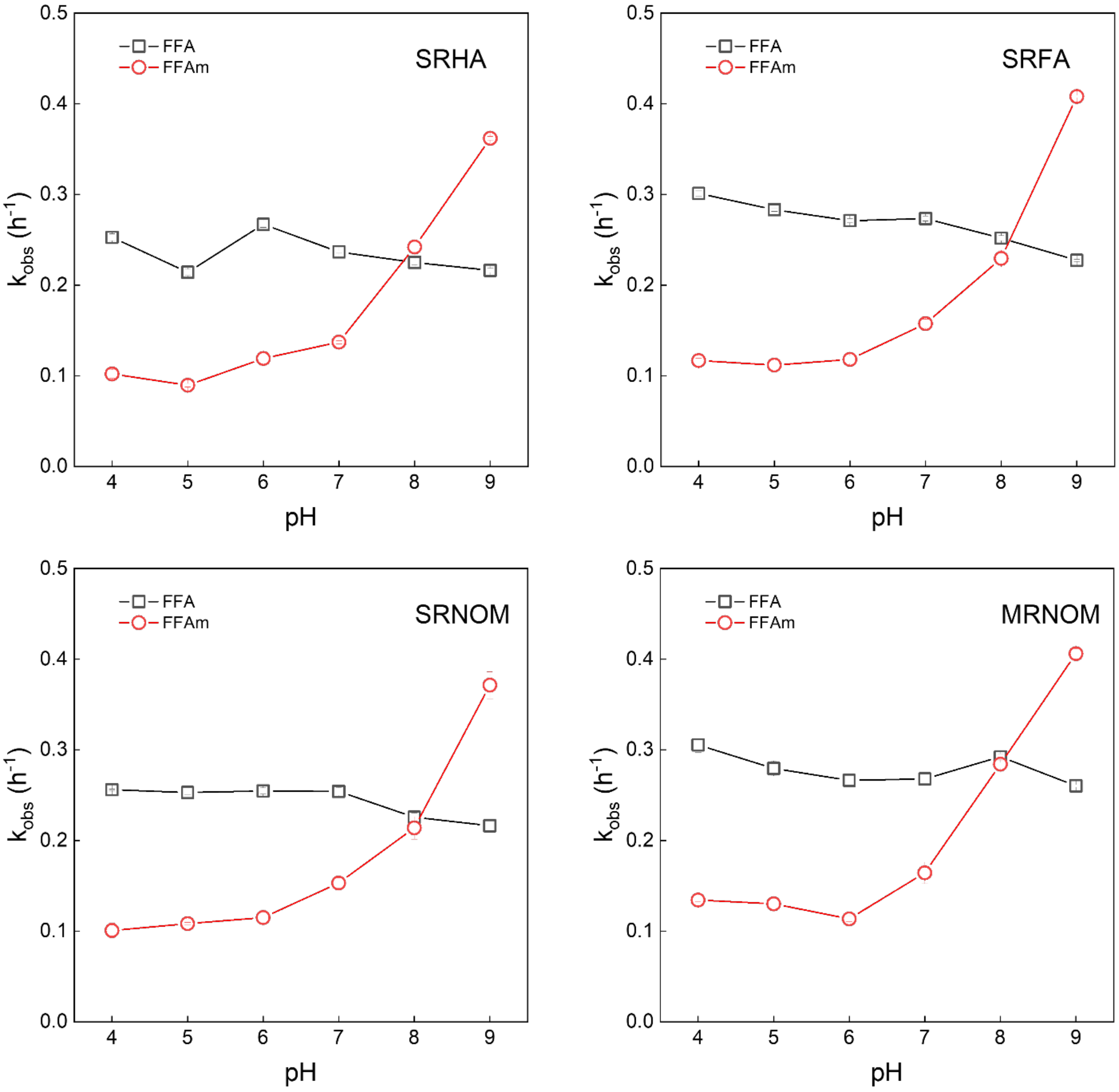
SRHA
SRFA
SRNOM
MRNOM
FFA
FFAm
k_{obs} (h^{-1})
pH
0.0
0.1
0.2
0.3
0.4
0.5
4
5
6
7
8
9

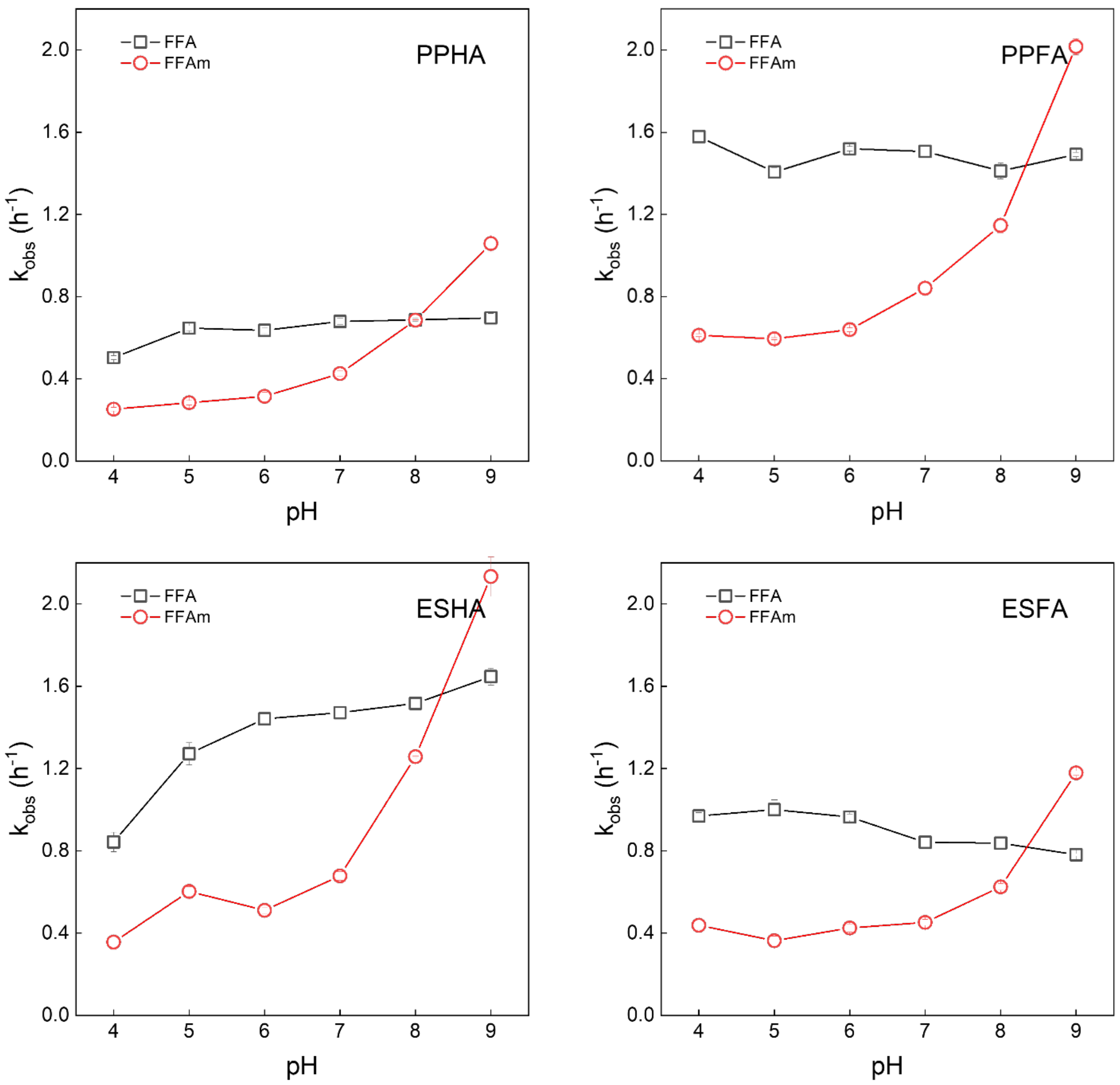

Figure S6. The observed first order rate constants of FFA and FFAm measured from different DOMs sensitized solutions at varying pHs. All DOM isolates were used as sensitizer at the concentration of 20 mg/L with FFA or FFAm added at added at 100 µM. Phosphate buffer (10 mM) was used to maintain the pH. Triplicates were conducted during the photolysis. The error bars indicated the standard deviation.

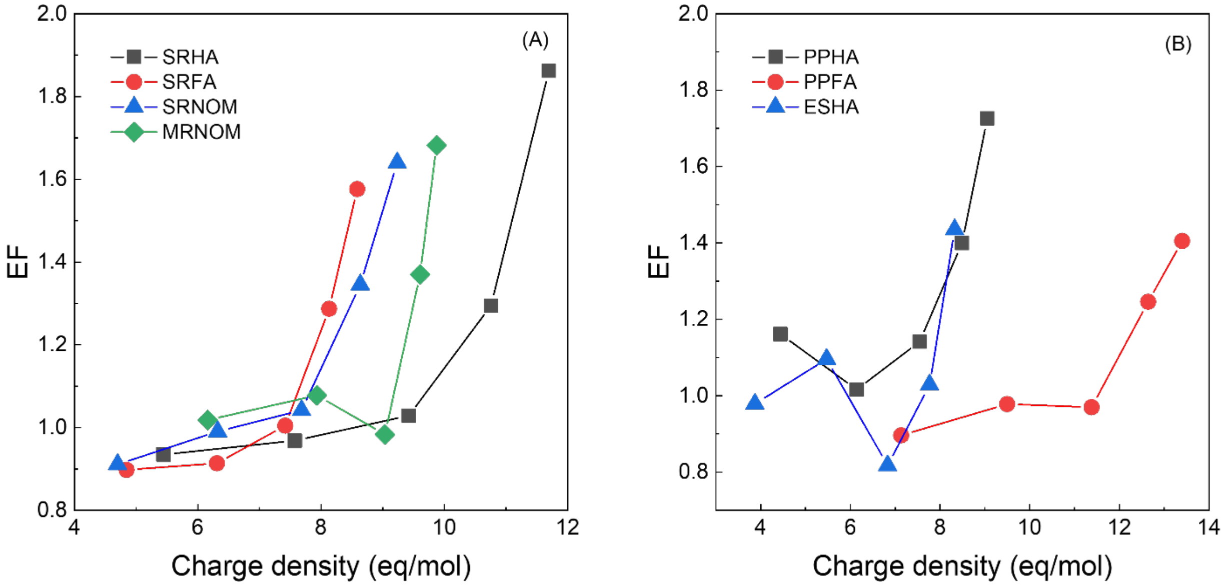

Figure S7. Enhancement factor plotted against the charge density for a variety of DOMs. EF was determined from photolysis experiments. Experimental conditions were detailed in method section. Charge density was calculated by Henderson-Hasselbalch modeling. Table S9 summarized the charge densities of each DOM isolate as a function of pH. (A) EF versus charge density for aquatic DOM isolates including SRHA, SRFA, SRNOM, and MRNOM. (B) EF versus charge density for soil DOM isolates including PPHA, PPFA, and ESHA.

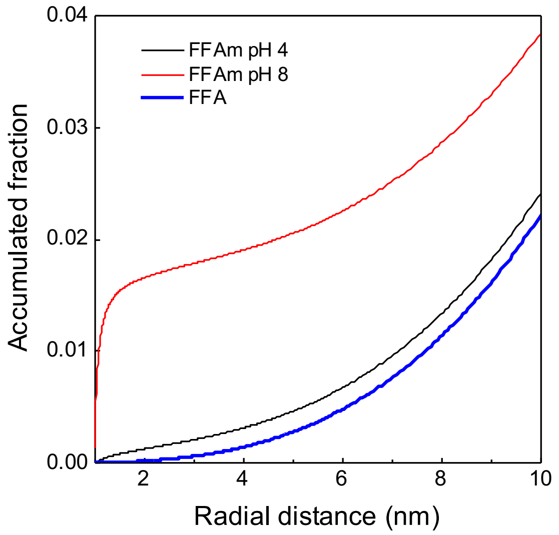

Figure S8. Cumulative fraction of FFAm (pH 4 and 8) and FFA in the vicinity of all SRHA molecules. The number of SRHA molecules was calculated using a concentration of 20 mg/L. the fraction distribution was determined from the solution to Poisson-Boltzmann equation (Text S7).

References

(1) Appiani, E.; Ossola, R.; Latch, D. E.; Erickson, P. R.; McNeill, K., Aqueous singlet oxygen reaction kinetics of furfuryl alcohol: effect of temperature, pH, and salt content. *Environ Sci-Proc Imp* **2017,** *19*, (4), 507-516.
(2) Rosario-Ortiz, F. L.; Canonica, S., Probe Compounds to Assess the Photochemical Activity of Dissolved Organic Matter. *Environ Sci Technol* **2016,** *50*, (23), 12532-12547.
(3) Williams, R.; Jencks, W.; Westheimer, F., pKa data compiled by R. Williams. In *University of Wisconsin-Madison,* https://www.chem.wisc.edu/areas/reich/pkatable/pKa_compilation-1-Williams.pdf 2019.
(4) Daza, M. C.; Doerr, M.; Salzmann, S.; Marian, C. M.; Thiel, W., Photophysics of phenalenone: quantum-mechanical investigation of singlet-triplet intersystem crossing. *Phys Chem Chem Phys* **2009,** *11*, (11), 1688-96.
(5) Benedetti, M. F.; vanRiemsdik, W. H.; Koopal, L. K., Humic substances considered as a heterogeneous donnan gel phase. *Environ Sci Technol* **1996,** *30*, (6), 1805-1813.
(6) Grandbois, M.; Latch, D. E.; Mcneill, K., Microheterogeneous Concentrations of Singlet Oxygen in Natural Organic Matter Isolate Solutions. *Environ Sci Technol* **2008,** *42*, (24), 9184-9190.
(7) Tipping, E.; Hurley, M. A., A Unifying Model of Cation Binding by Humic Substances. *Geochim Cosmochim Ac* **1992,** *56*, (10), 3627-3641.
(8) Latch, D. E.; McNeill, K., Microheterogeneity of singlet oxygen distributions in irradiated humic acid solutions. *Science* **2006,** *311*, (5768), 1743-1747.
